# How to improve infectious disease prediction by integrating environmental data: an application of a novel ensemble analysis strategy to predict HFMD

**DOI:** 10.1017/S0950268821000091

**Published:** 2021-01-15

**Authors:** Junwen Tao, Yue Ma, Xuefei Zhuang, Qiang Lv, Yaqiong Liu, Tao Zhang, Fei Yin

**Affiliations:** 1 West China School of Public Health and West China Fourth Hospital, Sichuan University, Chengdu, China; 2 Sichuan Center for Disease Control and Prevention, Chengdu, Sichuan, People's Republic of China

**Keywords:** Air pollutants, an ensemble prediction model, hand, foot and mouth disease, meteorological factors

## Abstract

This study proposed a novel ensemble analysis strategy to improve hand, foot and mouth disease (HFMD) prediction by integrating environmental data. The approach began by establishing a vector autoregressive model (VAR). Then, a dynamic Bayesian networks (DBN) model was used for variable selection of environmental factors. Finally, a VAR model with constraints (CVAR) was established for predicting the incidence of HFMD in Chengdu city from 2011 to 2017. DBN showed that temperature was related to HFMD at lags 1 and 2. Humidity, wind speed, sunshine, PM_10_, SO_2_ and NO_2_ were related to HFMD at lag 2. Compared with the autoregressive integrated moving average model with external variables (ARIMAX), the CVAR model had a higher coefficient of determination (*R*^2^, average difference: + 2.11%; *t* = 6.2051, *P* = 0.0003 < 0.05), a lower root mean-squared error (−24.88%; *t* = −5.2898, *P* = 0.0007 < 0.05) and a lower mean absolute percentage error (−16.69%; *t* = −4.3647, *P* = 0.0024 < 0.05). The accuracy of predicting the time-series shape was 88.16% for the CVAR model and 86.41% for ARIMAX. The CVAR model performed better in terms of variable selection, model interpretation and prediction. Therefore, it could be used by health authorities to identify potential HFMD outbreaks and develop disease control measures.

## Introduction

Prediction provides a better understanding and quantitative assessment for infectious disease control and risk evaluation. Accurate and explainable predictions also provide useful information for health administration and policymakers. Therefore, accuracy and interpretability are among the most important objectives of prediction research. With the establishment and development of the biopsychosocial model, most researchers pay attention not only to the infectious disease itself, but also to the impact of environmental factors, socio-economic factors, human behaviour and other factors on infectious disease prediction. In the past decade, due to climate change and environmental pollution, people have become increasingly concerned about the health effects of external environmental factors, such as temperature, relative humidity and air pollutants. Many of these factors have been found to have health effects and have become potential predictors in infectious disease prediction [1–3]. Introducing such external environmental factors into prediction models also improves the performance of the models. Basile *et al*. used meteorological data to predict the incidence rate of influenza and the accuracy of prediction was above 80% [4]. Additionally, Guo *et al*. used climate data to predict cases of dengue more accurately [5].

However, most external environmental factors affect infectious disease simultaneously and are thus usually correlated (e.g. temperature, humidity and rainfall). These correlation factors can cause multiple collinearities, hide the real relationship between the factors, even generate confounding correlation paths, thereby jeopardising the performance of the prediction model [6, 7]. Therefore, when using multiple correlated environmental factors (or multivariate time series, MTS) for prediction, how to introduce these factors into the model remains a major challenge.

Traditionally, the autoregressive integrated moving average model with external variables (ARIMAX) is commonly used to predict MTS data. However, the ARIMAX model often encounters various problems, such as variable selection and model interpretation. In this study, we proposed a novel ensemble analysis strategy to solve these problems and establish a prediction model within a unified framework. The ensemble analysis strategy started by establishing a vector autoregressive model (VAR) with all the external environment variables. Then, the VAR model was equivalently transformed into a dynamic Bayesian networks (DBN) model [8], and the latter was used to select the variables, which could be considered as a constraint condition. Finally, the VAR model with the constraint condition (CVAR) was established for prediction. Our previous studies proved that the VAR model can accurately predict MTS data and can be interpreted by impulse response analysis [9]. Moreover, DBN can identify the correlation among multiple variables simultaneously [10], while common correlation analysis can only identify two variables at a time. The variable selection process of DBN can simulate the real-world context in which influencing factors impact diseases. Based on these advantages, this study combined the two models under a unified mathematical framework to construct CVAR and improve the accuracy and interpretability of the prediction model.

In this study, hand, foot and mouth disease (HFMD) was used as an example to illustrate this ensemble analysis strategy, and the prediction performance was compared with that of a commonly used prediction model, the ARIMAX model, to evaluate the prediction accuracy and interpretability of the proposed strategy. HFMD is a major public health problem in China that is caused mainly by enterovirus 71 (EV71) and coxsackievirus A16 (CVA16) [11]. Many studies have found that environmental factors are related to HFMD incidence. For example, the relationship of temperature and relative humidity with HFMD approximated a positive linear association, while that of air pressure approximated a negative linear association [12, 13]. In the following section, the step-by-step application of our proposed ensemble analysis strategy was presented in the context of HFMD incidence prediction.

## Material and methods

### Study area and data sources

Our previous studies in Chengdu city found that temperature, diurnal temperature range and particulate matter under 10 μm (PM_10_) are related to HFMD [14–16]. Therefore, this study selected Chengdu city as the study area and included more environmental factors to conduct prediction research. Chengdu is the capital city of Sichuan Province, which lies in the west of the Sichuan Basin and at the centre of the Chengdu Plains. Air pollution in Chengdu is relatively serious because of the basin terrain. Chengdu lies in the subtropical humid climate subzone under the eastern-monsoonal region. The annual average temperature is 16.5 − 18.0°C, the annual maximum and minimum temperatures are 35.2 − 37.4°C and −5.3 ~ −1.4°C, respectively, and the annual average precipitation is 800 − 1400 mm. According to our previous studies and a meta-analysis, these climatic features could increase the risk of HFMD [14–17].

This study collected HFMD surveillance data and 10 environmental factors to develop prediction models. Daily HFMD data among children aged 0–14 years, from 1 January 2011 to 31 December 2017, were obtained from the Sichuan Center for Disease Control and Prevention (https://www.sccdc.cn/). All HFMD cases were confirmed by clinical diagnosis and met the National Guideline on Diagnosis and Treatment of Hand Foot Mouth Disease.

Environmental factors included meteorological factors and air pollutants. Daily meteorological data from 2011 to 2017 were obtained from the China National Weather Data Sharing System (http://data.cma.cn/). Daily air pollutant data during the same period were obtained from the Sichuan Environmental Monitoring Center (http://sthjt.sc.gov.cn/sthjt/c104334/scemc.shtml). The names and abbreviations of the variables are shown in Table 1.

**Table 1.** Variable names, abbreviations and units in this study

Names	Abbreviations	Units
HFMD incidence	HFMD	1/1 000 000 day
Mean wind speed	WIN	km/h
Sunshine duration	SUN	h
Mean air pressure	PRES	kPa
Mean temperature	TM	°C
Precipitation	RAIN	mm
Diurnal temperature range	DTR	°C
Relative humidity	HUMID	%
Particulate matter under 10 μm	PM_10_	μg/m^3^
Sulphur dioxide	SO_2_	μg/m^3^
Nitrogen dioxide	NO_2_	μg/m^3^

*Note.* All the mean of environmental variables is a daily mean.

### Steps of the ensemble analysis strategy

#### Step 1. Data preparation

**Fig. 1.**
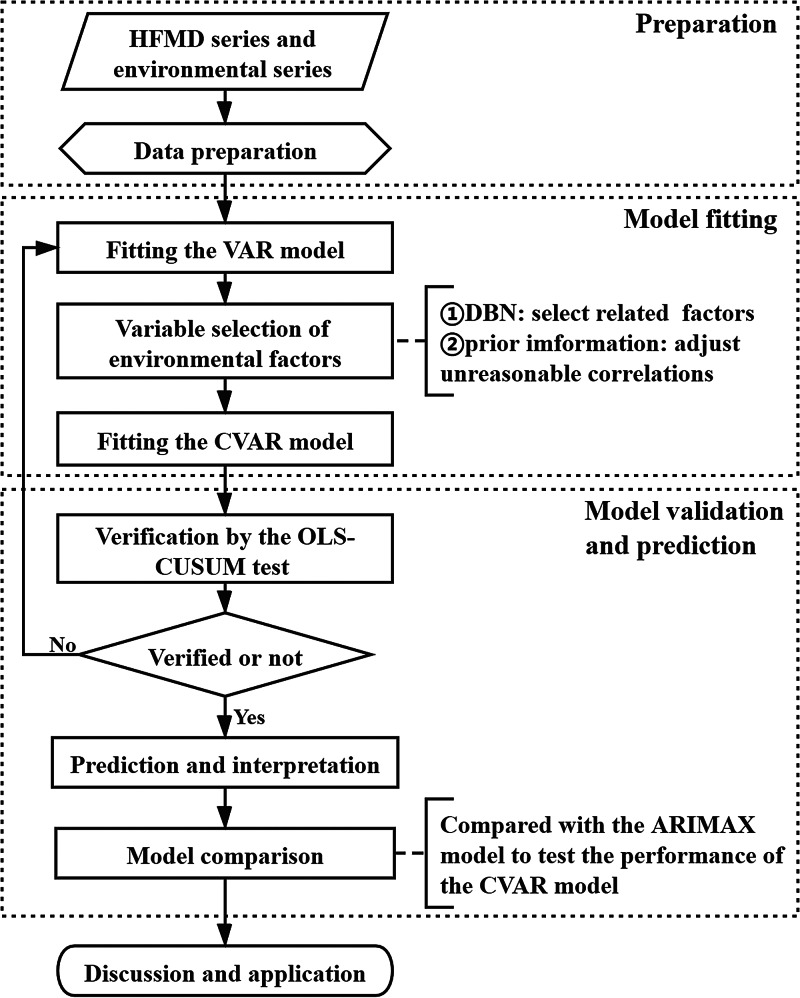
Process of the novel ensemble analysis strategy.

Figure 1 shows the process of the novel ensemble analysis strategy. Before fitting the VAR model, the trace statistic of the Johansen cointegration test was used to test a long-term equilibrium relationship of the MTS to avoid spurious regression (i.e. the random trend of several time series is the same). In addition, according to the stationary data requirement in time-series analysis and prediction [18], the augmented Dickey−Fuller (ADF) test was used to estimate the stationarity of the MTS: non-stationary MTS should be transformed by differencing to induce stationarity.

#### Step 2. The incorporation of environmental variables

The ensemble analysis strategy started by establishing a VAR model with all the external environment variables as follows:
1
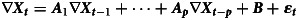


In equation (1), 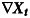 is the first-order differenced series of 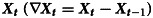, which represents environmental factors and HFMD incidence, and ***A_p_*** represents the coefficient matrix of the variables. Maximum likelihood estimation (MLE) was used to estimate the VAR parameters. ***B*** represents the baseline measurement for each variable; ***ɛ****_t_* is the residual series; *p* (1 ≤ *p* < *t*) is the maximum lag order, which represents the delayed effects of factors on HFMD and was determined by the Schwarz criterion (SC) via the ‘VARselect()’ function in the ‘vars’ package in R3.6.3. This maximum lag order was also used in the DBN.

#### Step 3. The selection of variables and the constraints on the VAR model

The DBN, which was used to select variables, is a directed acyclic graph that uses nodes and arcs to express the joint probability distribution function between variables. Nodes represent candidate variables in this study, and arcs (or arrows) represent the correlation between variables. For example, the correlation between nodes *i* and *j* could be measured by the coefficient *a_ij_* in ***A_p_*** (Eq. 1), where a larger *a_ij_* indicated a stronger correlation. Under such circumstances, the aim of variable selection via DBN is to identify whether *a_ij_* is non-zero for any two nodes *i* and *j* in the candidate set. In other words, if *a_ij_* is non-zero, then the network includes an arc between nodes *i* and *j*. The coefficients of DBN can be estimated by the least absolute shrinkage and selection operator (lasso), which uses a penalty term to constrain the sum of absolute parameter coefficients and shrink some coefficients to zero. In addition, variables selected by DBN usually have different lag orders, in contrast to Pearson's correlation analysis and other variable selection methods. However, the VAR model requires the same lag order for all variables; therefore, we propose the constrained VAR model (CVAR). In this model, the coefficients of unrelated variables in matrix ***A_p_*** (Eq. 1) were set to 0 by means of constraints that were obtained from DBN by the lasso method. The CVAR model is defined as follows:
2
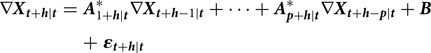

where 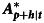 is the coefficient matrix with DBN constraints and *h* is the step of prediction, *h* ≥ 0. The other terms are the same as in equation (1). We used the ‘VAR()’ function in the ‘var’ package to fit the VAR models. After fitting the VAR models, we used the DBN and prior knowledge to impose constraints on the coefficient matrices and then used MLE to estimate the CVAR models. The OLS-CUSUM test was used to verify the stability over time of the coefficients of a linear regression model (i.e., CVAR) [19]. If the coefficients were within the confidence intervals, the models were considered effective and could be used for prediction.

#### Step 4. Model fitting and prediction

To make full use of the daily data, we conducted rolling training on the time-series data; that is, we took the three-year differenced data as a sample set (1094 days), analysed them in days and divided them into an integer training set (985 days) and test set (109 days) in a ratio of 9:1. Then, we scrolled forward the sample set in half a year until the end of the data. For example, the data from 1 January 2011 to 31 December 2013 were the first sample set. We divided this sample set into a training set and test set at a ratio of 9:1. The data from 1 July 2011 to 30 June 2014 were the second sample set and were also divided by a ratio of 9:1. In this way, we obtained nine sample sets and fitted nine VAR models (referred to as VAR_①−⑨). The nine sample sets were also used to fit the DBN, CVAR and ARIMAX models (referred to as DBN_①−⑨, CVAR_①−⑨ and ARIMAX_①−⑨). The coefficient of determination (*R*^2^) was used to evaluate the proportion of the variance explained and the goodness of fit for these models.

In addition, we summarised the graphs of the DBN_①−⑨ in one graph to show the results briefly. Based on the voting principle, the arcs of each variable within the DBN_①−⑨ appearing at least six times (more than half the times) were included in the summarised DBN graph. The corresponding coefficients were the average coefficients of these arcs.

The HFMD surveillance data can be updated within 24 h, thus, a dynamic prediction method was used to predict the incidence of HFMD 1-day ahead [20]. In addition, the results of 2-, 3-, 7-, 10-days ahead dynamic prediction and direct prediction without updating the data (109-days ahead) can be referred to Supplementary File, Tables S7 and S8. The dynamic prediction with 1-day ahead is that after one day of out-of-sample prediction, the training set was updated with observed data, the prediction model was re-fitted, and the re-fitted model was used to make the next 1-day ahead out-of-sample prediction. This process was repeated until the complete test set was predicted. The root mean-squared error (RMSE) and mean absolute percentage error (MAPE), which quantified the error between the actual and predicted values, were used to evaluate the prediction accuracy. A confusion matrix was used to summarise the ability of the CVAR model to predict increases and decreases in nine subsets [21]. Then, we averaged the results of the subsets and estimated the average accuracy.

#### Step 5. The enhancement of interpretability

*Step 5.1 Sensitivity analysis*: The sensitivity analysis was used to evaluate the importance of related variables selected by the summarised DBN graph in predicting the incidence of HFMD. This analysis was conducted based on the deletion of an environmental factor from the full CVAR model, and then the RMSE was calculated to check whether this factor could affect the prediction of HFMD incidence.

*Step 5.2 Impulse response analysis*: After establishing the CVAR model, the interpretability of the model was enhanced by the impulse response analysis, which is based on the Wold moving average function [18], and the model structure is as follows:
3
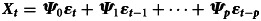

where ***Ψ***_***p***_ is the coefficient matrix of the impulse response. The ‘irf()’ function in the ‘var’ package was used to conduct the impulse response analysis to evaluate the response of a dependent variable in the next 10 days when an independent variable was subject to an impulse (changed by a unit). Because the average incubation period of HFMD is three to seven days, the period of 10 days could reflect the effects of environmental variables on HFMD. This analysis helped to explain the dynamic effects of predictors on response variables; therefore, we used it to enhance the interpretability of the CVAR model. The impulse response analysis was performed on each CVAR_①−⑨ model, and the variables that appeared in the summarised DBN graph were extracted. The impulse response results of these variables were averaged to construct a summarised impulse response analysis.

#### Step 6. Model comparison

To verify the performance of the CVAR model, we compared it with the ARIMAX model. The ARIMAX model is a classic method in prediction research, which provides a general analysis framework for predicting infectious disease [22]. Since exogenous variables need to be introduced and ARIMAX is a linear model, researchers usually use Pearson's correlation analysis to select variables and then fit the prediction model [23]. MLE was used to estimate the parameters of ARIMAX, and the optimal ARIMAX models were selected based on the Akaike information criterion (AIC) via the ‘auto.arima()’ function in the ‘forecast’ package in R3.6.3. The Ljung−Box test was used to verify the stability of ARIMAX models. When *P*_Ljung−Box_ > 0.05, indicating that the residual is white noise and the model is effective for prediction.

The *R*^2^, RMSE, MAPE and averaged confusion matrix of the ARIMAX models were estimated and compared with those of the CVAR models. A two-tailed paired *t*-test was used to test whether the *R*^2^, RMSE and MAPE of the two models were different. The ranges of *R*^2^, RMSE and MAPE were calculated to reflect the stability of the two models.

All the above statistical analyses were performed in R 3.6.3 using packages such as ‘bnlearn’, ‘lars’, ‘vars’, ‘tseries’ and ‘forecast’.

## Results

**Fig. 2.**
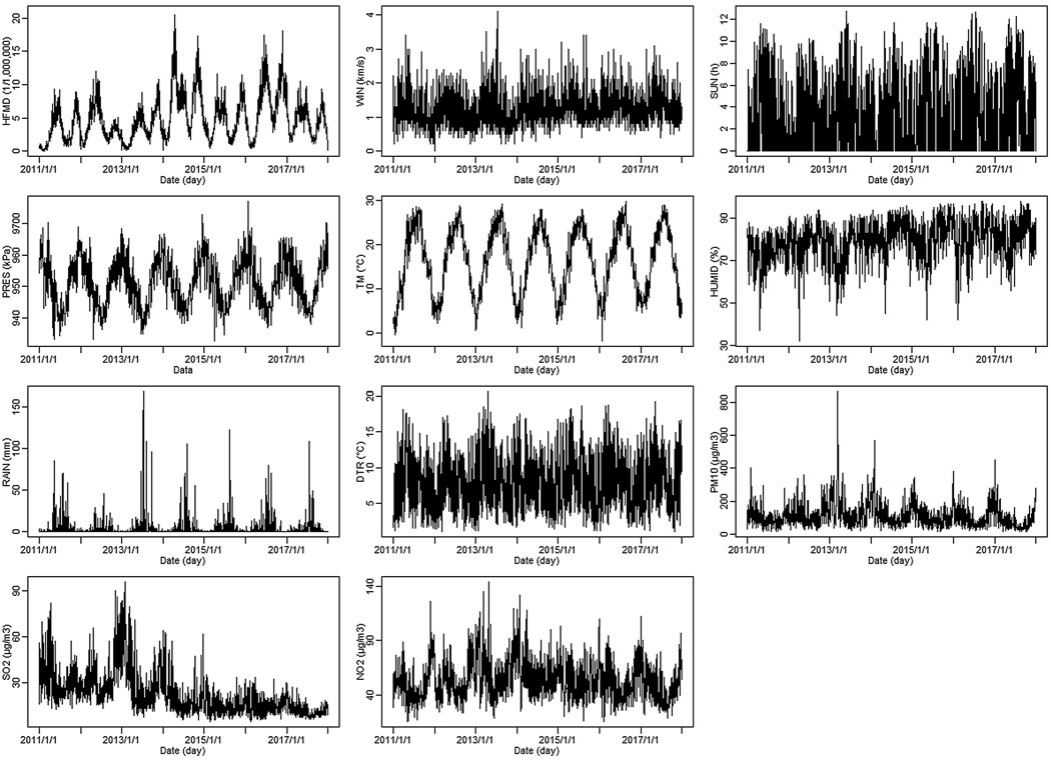
Time-series plots of variables in this study.

From 1 January 2011 to 31 December 2017, a total of 184 210 cases of HFMD were reported among children aged 0−14 years in Chengdu. The incidence rates were about 5 cases per 10 00 000-person day in Chengdu. Figure 2 shows the time-series plots of all the variables and Table 2 shows the statistical descriptions.

**Table 2.** Descriptions of daily HFMD incidence, meteorological and air pollution variables in Chengdu from 2011 to 2017

Variables	Mean	s.d.	Min.	Median	Max.
HFMD (1/1 000 000 day)	4.87	10.68	0.00	4.33	20.47
WIN (km/h)	1.22	0.48	0.00	1.15	4.10
SUN (h)	2.73	3.33	0.00	1.00	12.70
PRES (kPa)	951.00	7.43	932.50	950.90	977.00
TM (°C)	16.45	7.40	−1.90	17.40	29.80
HUMID (%)	79.35	8.64	32.00	80.00	98.00
RAIN (mm)	2.65	9.56	0.00	0.00	167.60
DTR (°C)	7.98	3.86	1.00	7.60	20.60
PM_10_ (μg/m^3^)	111.90	71.42	15.00	94.00	862.00
SO_2_ (μg/m^3^)	21.98	13.79	4.00	18.00	96.00
NO_2_ (μg/m^3^)	53.35	17.98	15.00	50.00	144.00

This study included 11 time series, and we conducted the Johansen cointegration test on the original data first. For all the *r* values tested, there were at least 11 cointegration ranks (Supplementary File, Table S1). Thus, the original data have a long-term equilibrium relationship and would not undergo spurious regression. The ADF statistic values showed that TM and HFMD were non-stationary, which might be related to the long-term time variation of these variables, thus we performed a first-order differencing for all the data (Supplementary File, Table S2). Then, we used the differenced data to establish the VAR, DBN, CVAR and ARIMAX models. Finally, the data were converted to the original scale.

### The parameter estimates of the DBN and CVAR models

**Fig. 3.**
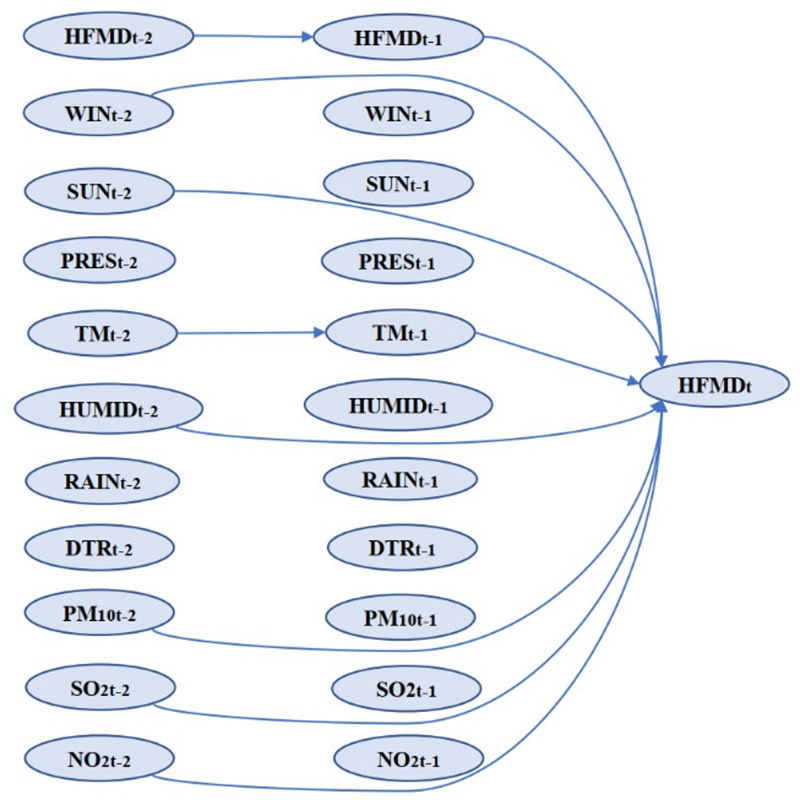
Summarised DBN graph of the DBN_①−⑨ models.

We performed rolling training on the MTS data. According to the Schwarz criterion (SC) values of the nine different training sets, the optimal maximum lag orders were two (Supplementary File, Table S3), and VAR_①−⑨ models were fitted with maximum lag = 2 (Supplementary File, VAR_①−⑨ Equations). Then, DBN_①−⑨ models were used to select the variables of the VAR_①−⑨ models. The parameters of the DBN_①−⑨ models are shown in the Supplementary File, Table S4 and Figure S1. In the nine DBN graphs, arcs appearing at least six times (more than half the times) were included in the summarised DBN graph (Fig. 3). The corresponding coefficients are shown in the Supplementary File, Table S5. The factors' coefficients ≠ 0 had arc connections, indicating correlations to HFMD, while coefficients = 0 would not show in the summarised DBN graph and were not related to HFMD. TM was related to HFMD at both lag 1 and lag 2, indicating that TM was an important factor for HFMD.

HFMD has no impact on environmental factors. Therefore, in the VAR models with environmental factors as dependent variables and HFMD as an independent variable, the coefficient of HFMD was zero. Combined with the variables selected by DBN, we imposed two types of constraints on the VAR models (DBN variable selection and common knowledge). We followed this approach to fit the CVAR_①−⑨ models. The coefficients of the CVAR models can be found in the Supplementary File, CVAR_①−⑨ Equations. The results of the OLS-CUSUM test indicated that all the coefficients were within the confidence intervals, and the CVAR_①−⑨ models were stable and effective (Supplementary File, Fig. S2).

### The interpretation of the CVAR model

#### Sensitivity analysis

According to the summarised DBN graph, we deleted a related factor from the full CVAR model once a time and compared the increased RMSE of each model (Table 3). The sensitivity analysis results showed that temperature, wind speed and humidity had great effects on the HFMD prediction. NO_2_ and PM_10_ were important predictors among the air pollutants.

**Table 3.** The sensitivity analysis of the CVAR_①−⑨ models

Model	RMSE							
	Full model	-WIN	-SUN	-TM	-HUMID	-PM_10_	-SO_2_	-NO_2_
①	1.066	1.0751	1.0750	1.0754	1.0719	1.0709	1.0750	1.0799
②	1.072	1.0809	1.0870	1.0804	1.0881	1.0871	1.0710	1.0858
③	1.145	1.1448	1.1444	1.1438	1.1524	1.1518	1.1440	1.1568
④	1.147	1.1468	1.1468	1.1453	1.1548	1.1543	1.1464	1.1386
⑤	1.151	1.1595	1.1501	1.1596	1.1478	1.1475	1.1495	1.1524
⑥	1.149	1.1480	1.1485	1.1471	1.1464	1.1457	1.1478	1.1395
⑦	1.148	1.1451	1.1469	1.1467	1.1446	1.1439	1.1463	1.1382
⑧	1.147	1.1440	1.1460	1.1561	1.1438	1.1431	1.1475	1.1475
⑨	1.140	1.1438	1.1394	1.1512	1.1374	1.1353	1.1414	1.1410
Average	1.129	1.1320	1.1316	1.1339	1.1319	1.1311	1.1299	1.1311
Icrease(%)	\	0.2416	0.2028	0.4133	0.2331	0.1579	0.0529	0.1588

*Note.* ‘-’ represents the deletion of this variable from the full model.

#### Impulse response analysis

**Fig. 4.**
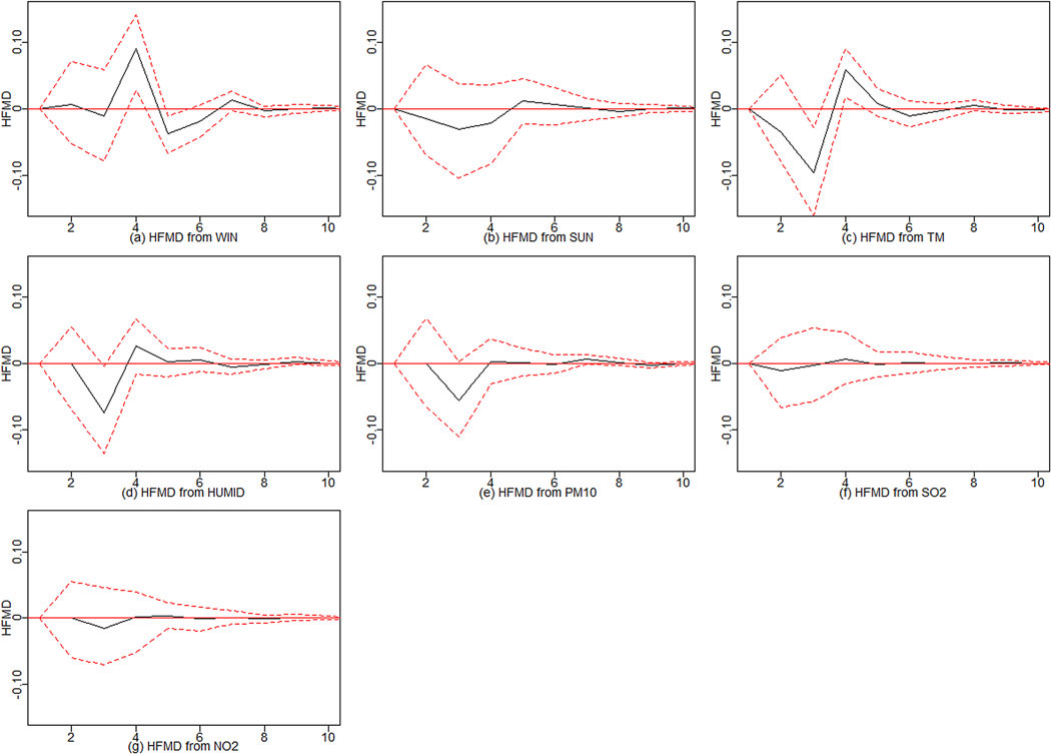
Summarised impulse response analysis of the CVAR_①−⑨ models.

After establishing CVAR_①−⑨ models, we estimated the effects of environmental variables on HFMD through the impulse response analysis. Figure 4 shows a summary of the impulse response analysis. Wind speed was positively related to HFMD during the first five days, negatively related to HFMD on days five to seven and then tended to be zero. Sunshine was negatively related to HFMD during the first five days, positively related on days five to seven and finally tended to be zero. Temperature was negatively related to HFMD during the first three days, positively related to HFMD on day four and day five and then tended to be zero. Humidity was negatively related to HFMD during the first four days, positively related to HFMD on the next several days, and then tended to be zero. PM_10_, SO_2_ and NO_2_ were negatively related to HFMD during the first three or four days and then had a positive impact, eventually tending to zero.

### The results of model comparison

On the basis of the Pearson correlation analysis conducted on the nine training sets (Supplementary File, Table S6), we selected the relevant environmental variables and used them to fit ARIMAX_①−⑨ models (Table 4). The Ljung−Box test showed that the residuals of each model were white noise; therefore, the ARIMAX_①−⑨ models were stable and could be used for prediction.

**Table 4.** Results of the ARIMAX_①−⑨ models

Model	Varibles	AIC	*P*_Ljung−Box_
①	ARIMA(4,0,3) + SUN,PRES,TM,DTR,PM_10_,SO_2_	2289.820	0.998
②	ARIMA(5,0,2) + PRES,TM,DTR,PM_10_,SO_2_,NO_2_	2415.170	0.947
③	ARIMA(5,0,5) + PRES,TM,HUMID,PM_10_,SO_2_	2711.660	0.946
④	ARIMA(3,0,3) + PRES,TM,HUMID,PM_10_,SO_2_	2822.610	0.988
⑤	ARIMA(2,0,3) + TM,HUMID,DTR, PM_10_,SO_2_,NO_2_	2905.470	0.981
⑥	ARIMA(5,0,3) + PRES,TM,PM_10_	2906.230	0.960
⑦	ARIMA(2,0,4) + WIN,PRES,TM,HUMID,PM_10_,SO2,NO2	3042.750	0.984
⑧	ARIMA(5,0,4) + WIN,PRES,TM,HUMID,DTR, PM_10_	2989.740	0.515
⑨	ARIMA(5,0,4) + WIN,PRES,TM,HUMID	2868.450	0.510

**Fig. 5.**
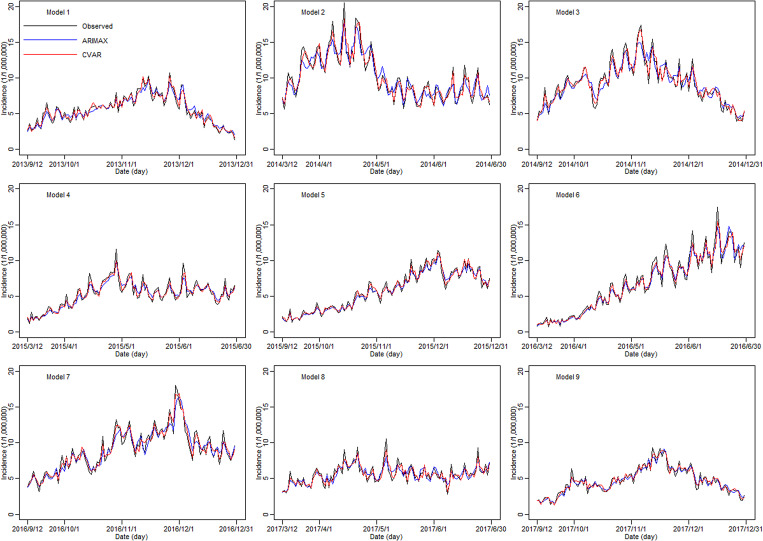
Incidence of HFMD predicted by the CVAR_①−⑨ and ARIMAX_①−⑨ models in the test set.

**Fig. 6.**
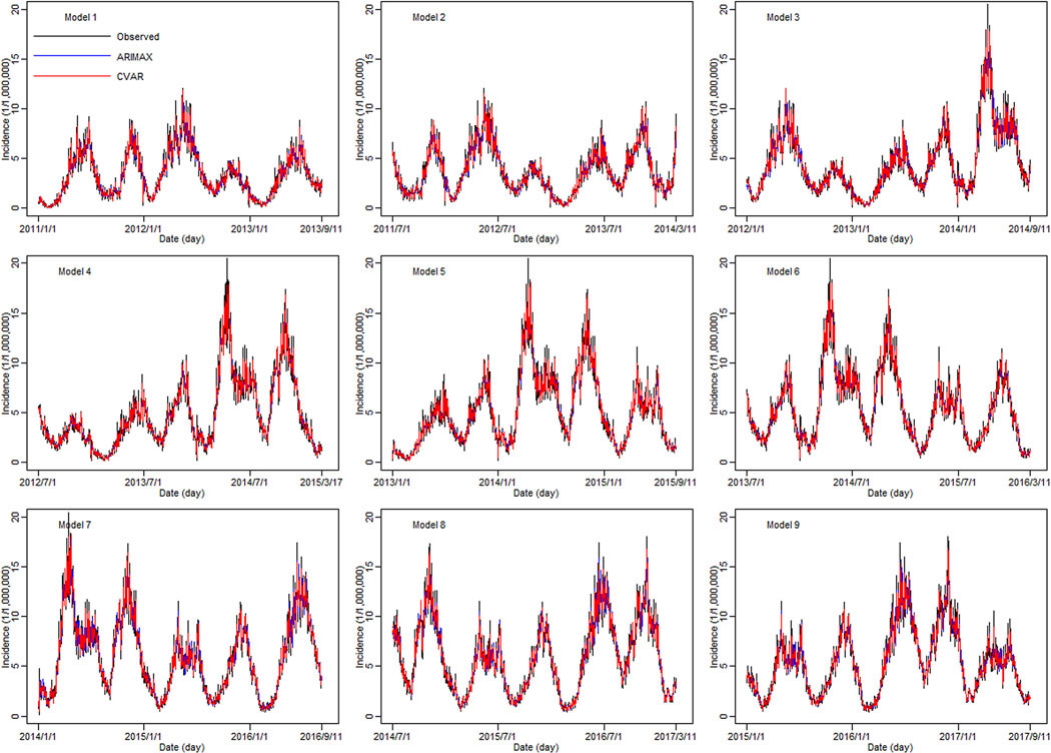
Incidence of HFMD fitted by the CVAR_①−⑨ and ARIMAX_①−⑨ models in the training set.

In the training sets, the coefficients of determination (*R*^2^) were calculated for CVAR_①−⑨ and ARIMAX_①−⑨ models. In the test set, the CVAR and ARIMAX models were used to predict the incidence of HFMD 1-day ahead (Table 5). The results of 2, 3, 7, 10-days ahead dynamic prediction and direct prediction (109-days ahead) can be referred to Supplementary File, Tables S7 and S8. Compared with the ARIMAX model, the CVAR model showed a significantly higher *R*^2^ (average difference: +2.11%; two-tailed paired *t*-test: *t* = 6.2051, *P* = 0.0003 < 0.05), a lower RMSE (−24.88%; *t* = −5.2898, *P* = 0.0007 < 0.05) and a lower MAPE (−16.69%; *t* = −4.3647, *P* = 0.0024 < 0.05). These indicated that the CVAR models performed better than the ARIMAX models. The ranges of *R*^2^, RMSE and MAPE of the CVAR models were always narrower than those of the ARIMAX models, indicating that the performance of the CVAR model was more stable. Figures 5 and 6 show the prediction and fitting plots, respectively. Comparison of the averaged confusion matrices of the two models (Tables 6 and 7) indicated that the accuracy of the CVAR models was 88.16% ((50.26 + 45.84)/109) and that of the ARIMAX models was 86.41% ((48.56 + 45.63)/109). Thus, the CVAR models were more accurate in predicting the time-series shape.

**Table 5.** Comparisons of *R*^2^, RMSE, MAPE, ranges and means between the CVAR_①−⑨ and ARIMAX_①−⑨ models for 1-day ahead dynamic prediction

Model	*R*^2^		RMSE		MAPE(%)	
	CVAR	ARIMAX	CVAR	ARIMAX	CVAR	ARIMAX
①	0.963	0.931	1.066	1.221	100.69	113.40
②	0.958	0.921	1.072	1.852	99.89	140.15
③	0.969	0.951	1.145	1.699	99.61	140.60
④	0.972	0.951	1.147	1.357	100.19	124.61
⑤	0.973	0.951	1.151	1.451	99.28	124.09
⑥	0.968	0.951	1.149	1.552	100.04	100.70
⑦	0.969	0.952	1.148	1.690	99.15	112.32
⑧	0.970	0.962	1.147	1.340	99.81	112.20
⑨	0.966	0.958	1.140	1.370	100.20	110.86
Range	(0.958, 0.973)	(0.921, 0.962)	(1.066, 1.151)	(1.221, 1.825)	(99.15, 100.69)	(100.7, 140.6)
Mean	0.968	0.948	1.129	1.503	99.87	119.88

**Table 6.** The averaged confusion matrix of the CVAR models

Actual	CVAR predicting		Total
	Up	Down	
Up	50.26	5.62	55.88
Down	7.28	45.84	53.12
Total	57.54	51.46	109

**Table 7.** The averaged confusion matrix of the ARIMAX models

Actual	ARIMAX predicting		Total
	Up	Down	
Up	48.56	8.25	56.81
Down	6.56	45.63	52.19
Total	55.12	53.88	109

## Discussion

Accuracy and interpretability have always been among the most important objectives of infectious disease prediction. In this study, data on environmental factors and HFMD incidence in Chengdu city from 2011 to 2017 were used to establish CVAR models using an ensemble analysis strategy, and the prediction accuracy and interpretability of the models were evaluated. In this ensemble analysis strategy, we first established the VAR model, then used the DBN model to select variables under the unified framework and finally established the CVAR model. We found that the ensemble analysis strategy had advantages in terms of variable selection, model interpretation and prediction.

The DBN used in this strategy had advantages in variable selection and results display. The variables selected by the DBN (Fig. 3) included temperature, which was related to HFMD at lag 1 and lag 2, as well as relative humidity, wind speed, sunshine, PM_10_, SO_2_ and NO_2_, which were related to HFMD at lag 2. Previous work in Sichuan province showed that the meteorological factors, including temperature, humidity, sunshine, air pressure and wind speed, were related to HFMD [24], which is consistent with the results of our study. Many other works have provided several postulations to explain the pathways through which meteorological factors affect HFMD [25, 26]. One possible explanation is that meteorological factors could influence the transmission and the survival of the HFMD virus, as well as human behaviours, thereby influencing infection transmission. Several studies have found that air pollutants can increase the risk of respiratory diseases [27, 28]. One of the infection pathways of HFMD is the respiratory transmission. This might provide a possible explanation for why pollutants affect HFMD; however, more studies are needed to evaluate the mechanisms. A recent analysis of ours in Chengdu found that PM_10_ in air pollutants increased the risk of HFMD [16], and another study in Hefei found a significant statistical correlation between SO_2_ and HFMD [29], which could support the results of our present study. In addition, this study first found that NO_2_ had an impact on HFMD. This impact may be related to the high concentration of NO_2_ in Chengdu city and more studies are needed to test this finding. By contrast, other studies have found that wind speed, air pressure, SO_2_ and NO_2_ were not related to HFMD [30, 31]. Possible explanations for these discrepancies could be the different analysis methods and the differences in climatic and geographic conditions of the study areas.

This ensemble analysis strategy applied sensitivity analysis and impulse response analysis to enhance the interpretability of CVAR models. We found that temperature, wind speed and humidity had great effects on the HFMD prediction. NO_2_ and PM_10_ were more important among the air pollutant predictors. Temperature, humidity, sunshine, PM_10_, SO_2_ and NO_2_ were negatively related to HFMD during the first three or four days and then had a positive impact on the next three days, with the effect eventually tending to zero. This process is very similar to the clinical course of HFMD, which has an average incubation period of 3–7 days. This phenomenon may be attributed to the delayed effect of environmental factors on health [32]. Other studies found similar effects of temperature, humidity and air pollutants on HFMD [33, 34]. Wind speed was positively related to HFMD during the first several days, negatively related during the next few days, then positively related and finally unrelated, but the general effects were positive. These findings are supported by previous studies, which indicated that wind speed could increase the risk of HFMD [33]. However, our findings differ from other studies, which found that wind speed and sunshine have no statistically significant effect on HFMD [30]. Possible explanations for the discrepancies could be the differences in the environmental and socio-economic profiles of these study areas.

Compared with that of the VAR model, the structure of the CVAR model was more reasonable, which further improved the interpretability of the CVAR model. The VAR model allows all variables to be either explanatory or response variables, which often leads to unreasonable relationships in the model. Take the dengue VAR model established by Goto et al. as an example [35]. When temperature was taken as a response variable and dengue incidence as an explanatory variable, the model indicated that the incidence of dengue impacted temperature. This result is clearly not consistent with an epidemiological causal relationship. In our ensemble analysis strategy, the CVAR model could use DBN and prior information to adjust unreasonable correlations between variables and optimise the model structure.

In addition, the ensemble analysis strategy also had advantages in prediction. We compared the performance of the CVAR model with that of the ARIMAX model, which is a traditional model for the prediction of MTS data. The CVAR model had a higher *R*^2^ (+2.11%; *t* = 6.2051, *P* = 0.0003 < 0.05), a lower RMSE (−24.88%; *t* = −5.2898, *P* = 0.0007 < 0.05) and a lower MAPE (−16.69%; *t* = −4.3647, *P* = 0.0024 < 0.05). The ranges of *R*^2^, RMSE and MAPE of the CVAR models were always narrower than those of the ARIMAX models. These results suggested that the CVAR models could predict HFMD more accurately and stably. Comparing the confusion matrices of the two type models (Tables 6 and 7), the accuracy of the CVAR models was 88.16%, while the accuracy of the ARIMAX models was 86.41%, indicating that the CVAR models were more accurate in predicting the time-series shape. Besides, comparing the results of 1-, 2-, 3-, 7-, 10-days ahead dynamic prediction and direct prediction (109-days ahead), we found that the RMSE increased significantly after 7-days ahead and then reached a steady state. Therefore, it is better to set the prediction step within 7-days for HFMD prediction.

Based on this information, researchers can provide recommendations for health and related authorities to prevent and control HFMD. For example, according to the relationship between temperature, humidity, wind speed, sunshine, PM_10_, SO_2_, NO_2_ and HFMD, we recommend that the health sector establish a disease warning system based on meteorological variables. Furthermore, the meteorological department should warn of bad weather, especially when it is hot, stormy or hazy weather. The public should be reminded to take protective measures to reduce exposure to bad weather. Moreover, the government can reduce air pollution in the basin region by increasing green areas, promoting clean energy vehicles or encouraging citizens to install gas purification devices.

Under this novel framework, we used the VAR and DBN models to establish the CVAR models. This ensemble analysis strategy has some benefits. By using the DBN model to select variables, we can identify the complex correlation pattern and delayed effect among MTS data simultaneously, and the DBN can use the network graph to represent the relationship between variables by lag order, which is difficult to achieve in the ordinary correlation analysis. The characteristics of the DBN model make it applicable not only to HFMD prediction, but also to the prediction of other infectious diseases. Besides, common knowledge constraints made the structures of the CVAR models more reasonable, and the impulse response analysis enhanced the interpretability of the CVAR models. Furthermore, the *t*-tests of the *R*^2^, RMSE and MAPE showed that the CVAR models had a higher prediction accuracy. Additionally, the ranges of *R*^2^, RMSE and MAPE indicated that the CVAR models were more stable, and the averaged confusion matrix indicated that CVAR models could predict increases and decreases in the time series more accurately. The results of our study can provide useful recommendations for HFMD prevention and have a certain application value. Both the DBN and the ensemble analysis strategy have the potential to be applied in other infectious disease predictions.

However, some limitations require mentioning. First, our previous work suggested that at least three years of weekly HFMD data are required to fit the DBN model, as well as the CVAR model [10], while the ARIMAX model required four to seven seasonal cycles of data [18]. When the available HFMD surveillance data are insufficient, the parameters of these models might be unstable. Second, since the short-term dynamic prediction of a CVAR model is more accurate, surveillance data and prediction models must be updated constantly to ensure the accuracy of prediction. Third, the CVAR models analysed only environmental factors and did not include other field factors, such as socio-economic factors. Thus, more studies are needed to identify whether other factors could improve the prediction accuracy of CVAR models.

## Conclusion

In conclusion, the ensemble analysis strategy could accurately select variables and display the correlation pattern via a network graph. The interpretability and prediction accuracy of the CVAR models were better than those of the ARIMAX models. Health authorities can use the ensemble analysis strategy to identify potential HFMD outbreaks and apply this information to develop disease prevention and control measures.

## Data Availability

HFMD surveillance data were obtained from the Sichuan Center for Disease Control and Prevention (https://www.sccdc.cn/). Daily meteorological data were obtained from the China Meteorological Data Sharing Service System (http://data.cma.cn/). Daily air pollutant data were obtained from the Sichuan Environmental Monitoring Center (http://sthjt.sc.gov.cn/sthjt/c104334/scemc.shtml). Researchers who need these data can apply to these sectors on their websites and obtain the data after approval. Raw data will not be shared because the authors are not authorised to distribute the data.

## Supplementary material

For supplementary material accompanying this paper visit http://dx.doi.org/10.1017/S0950268821000091. click here to view supplementary material
