# Supplementary material for: How to improve infectious disease prediction by integrating environmental data: an application of a novel ensemble analysis strategy to predict HFMD
Source: Epidemiol Infect. 2021 Jan 15;149:e34. doi: 10.1017/S0950268821000091 (PMC8060825; doi:10.1017/S0950268821000091)
Supplement: Supplementary file 1 [file S0950268821000091sup001.pdf]

Epidemiology and Infection

How to improve infectious disease prediction by integrating environmental data: an application of novel ensemble analysis strategy to predict HFMD

Junwen Tao[1, #], Yue Ma[1, #], Xuefei Zhuang[1], Qiang Lv[2], Yaqiong Liu[2], Tao Zhang[1, *], Fei Yin[1, *]

[1] West China School of Public Health and West China Fourth Hospital, Sichuan University

[2] Sichuan Center for Disease Control and Prevention, Chengdu, Sichuan, People's Republic of China

[#] These authors contributed equally to this article and share first authorship

[*] Corresponding author: Tao Zhang, Fei Yin

Email: taozscu@163.com，scupublichealth@163.com

Supplementary Material

Table S1. The Johansen cointegration test

Hypothesis	Trace statistic	Critical value	*P* value
r≤10	15.07	16.26	0.01
r≤9	73.34	30.45	0.01
r≤8	298.55	48.45	0.01
r≤7	614.54	70.05	0.01
r≤6	951.56	96.58	0.01
r≤5	1347.29	124.75	0.01
r≤4	1958.78	158.49	0.01
r≤3	2733.36	196.08	0.01
r≤2	3561.66	234.41	0.01
r≤1	4506.74	279.07	0.01
r=0	5548.1	327.45	0.01

Table S2. The augmented Dickey-Fuller test and first-order differencing

Variables	ADF	*P* value	Result

HFMD	-3.5449	0.03796	Not stationary
D_HFMD	-13.675	0.01	Stationary
WIN	-9.4859	0.01	Stationary
D_WIN	-22.29	0.01	Stationary
SUN	-8.4461	0.01	Stationary
D_SUN	-19.616	0.01	Stationary
PRES	-4.1156	0.01	Stationary
D_PRES	-19.445	0.01	Stationary
TM	-2.8969	0.1986	Not stationary
D_TM	-15.756	0.01	Stationary
HUMID	-7.2008	0.01	Stationary
D_HUMID	-19.938	0.01	Stationary
RAIN	-8.9159	0.01	Stationary
D_RAIN	-21.282	0.01	Stationary
DTR	-8.7754	0.01	Stationary
D_DTR	-19.997	0.01	Stationary
PM10	-7.206	0.01	Stationary
D_PM10	-18.932	0.01	Stationary
SO2	-3.0328	0.1411	Not stationary
D_SO2	-19.662	0.01	Stationary
NO2	-4.9043	0.01	Stationary
D_NO2	-18.731	0.01	Stationary

Note. "D_" represents a first-order differencing.

Table S3. The Schwarz criterion (SC) of the VAR_①~⑨ models

	VAR Models								
	①	②	③	④	⑤	⑥	⑦	⑧	⑨
SC	30.673	31.976	30.463	30.478	31.543	29.754	27.745	28.865	27.176

Table S4. Coefficients of the DBN_①~⑨ models

Variables	DBN_①		DBN_②		DBN_③	
	Lag 1	Lag 2	Lag 1	Lag 2	Lag 1	Lag 2
HFMD	-0.177	-0.003	-0.187	-0.205	-0.206	-0.076
WIN	-0.094	-0.045	0.000	-0.053	0.000	0.000
SUN	-0.025	0.015	0.000	0.009	0.000	0.104
PRES	0.000	0.000	0.004	0.018	0.000	0.012
TM	-0.100	-0.081	-0.047	-0.159	-0.021	-0.174
HUMID	0.000	-0.010	0.000	-0.024	0.000	-0.016

RAIN	0.000	-0.003	0.000	-0.001	0.000	0.000
DTR	0.011	0.000	0.000	0.002	0.011	0.000
PM_{10}	0.000	0.000	0.000	-0.017	0.000	0.000
SO_2	0.000	-0.001	-0.101	0.000	-0.007	-0.005
NO_2	-0.001	0.000	0.000	-0.016	0.000	-0.021

Variables	DBN_④		DBN_⑤		DBN_⑥	
	Lag1	Lag2	Lag 1	Lag 2	Lag 1	Lag 2
HFMD	-0.305	-0.098	-0.313	-0.105	-0.300	-0.109
WIN	0.000	-0.006	0.000	-0.012	0.002	-0.002
SUN	0.000	0.014	0.000	0.011	0.000	0.013
PRES	0.000	0.000	0.045	0.042	0.015	0.033
TM	-0.041	-0.146	0.000	-0.201	-0.042	-0.110
HUMID	0.002	-0.003	0.000	-0.021	0.000	0.000
RAIN	0.000	0.000	0.000	-0.065	0.000	0.000
DTR	0.000	0.000	0.000	0.000	0.000	0.000
PM_{10}	0.000	-0.006	0.000	-0.010	0.000	-0.019
SO_2	-0.011	-0.011	-0.003	0.000	0.000	-0.003
NO_2	0.000	-0.017	0.000	-0.014	0.000	-0.012

Variables	DBN_⑦		DBN_⑧		DBN_⑨	
	Lag 1	Lag 2	Lag 1	Lag 2	Lag 1	Lag 2
HFMD	-0.212	-0.106	-0.298	-0.137	-0.315	-0.142
WIN	0.007	-0.003	0.014	-0.012	0.009	-0.011
SUN	0.000	0.006	0.009	0.000	0.001	0.001
PRES	0.021	0.044	0.000	0.000	0.000	0.000
TM	-0.002	-0.145	-0.060	-0.184	-0.047	-0.221
HUMID	0.000	0.000	-0.003	-0.007	-0.011	-0.017
RAIN	0.000	-0.002	0.000	-0.002	0.000	-0.006
DTR	0.000	0.000	0.000	0.000	0.000	0.000
PM_{10}	0.000	-0.018	0.000	-0.020	0.000	-0.009
SO_2	0.000	-0.006	0.005	-0.001	0.000	0.000
NO_2	0.000	0.000	0.000	0.000	-0.007	-0.014

Table S5. The averaged coefficients and its original coefficient ranges of the DBN_①~⑨ models which coefficients were nonzero at least six times

Varia-bles	Lag 1		Lag 2	
	Average coefficient	Range of original coefficient	Average coefficient	Range of original coefficient
HFMD	-0.257	(-0.315,-0.177)	-0.109	(-0.205,-0.003)
WIN	0	0	-0.016	(-0.053,0)
SUN	0	0	0.019	(0,0.104)

PRES	0	0	0	0
TM	-0.040	(-0.1,0)	-0.158	(-0.221,-0.081)
HUMID	0	0	-0.011	(-0.024,0)
RAIN	0	0	0	0
DTR	0	0	0	0
PM_{10}	0	0	-0.011	(-0.020,0)
SO_2	0	0	-0.003	(-0.011,0)
NO_2	0	0	-0.010	(-0.021,0)

Table S6. Pearson correlation analysis of the sample set_①~⑨

Sample set	Variables	WIN	SUN	PRES	TM	HUMID	RAIN	DTR	PM_{10}	SO_2	NO_2
①	HFMD	-0.06	0.10*	-0.20*	0.41*	0.04	0.02	0.09*	-0.25*	-0.19*	0.04
②	HFMD	0.02	0.11	-0.12*	0.19*	0.04	-0.02	0.08*	-0.15*	-0.29*	0.08*
③	HFMD	0.03	0.04	-0.12*	0.22*	0.23*	0.00	0.03	-0.20*	-0.37*	-0.03
④	HFMD	0.02	0.02	-0.16*	0.13*	0.18*	0.01	-0.10	-0.24*	-0.33*	-0.05
⑤	HFMD	-0.04	-0.02	0.01	0.13*	0.19*	-0.05	-0.12*	-0.23*	-0.28*	-0.11*
⑥	HFMD	-0.03	0.04	-0.08*	0.11*	0.04	-0.04	-0.00	-0.13*	-0.05	-0.05
⑦	HFMD	-0.07*	0.03	-0.32*	0.21*	0.31*	0.00	-0.04	-0.28*	-0.09*	-0.08*
⑧	HFMD	-0.07*	0.05	-0.12*	0.18*	0.21*	0.02	-0.07*	-0.14*	0.01	-0.04
⑨	HFMD	-0.08*	0.06	-0.22*	0.13*	0.07*	-0.01	-0.01	-0.03	0.04	-0.03

Note. Statistically significant ($P < 0.05$) were labelled with an asterisk.

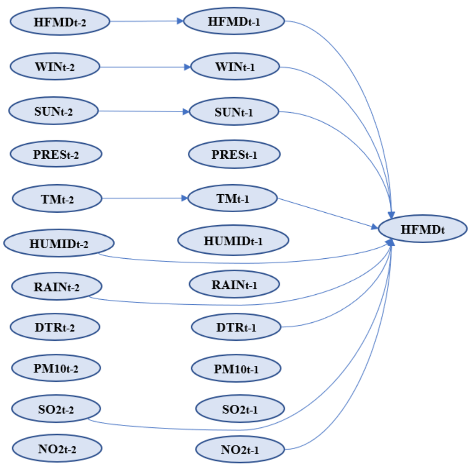

DBN_①

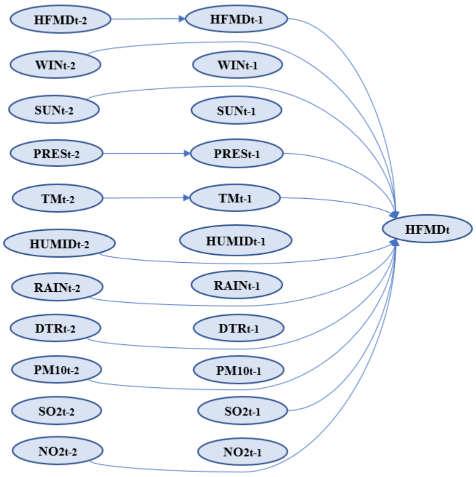

DBN_②

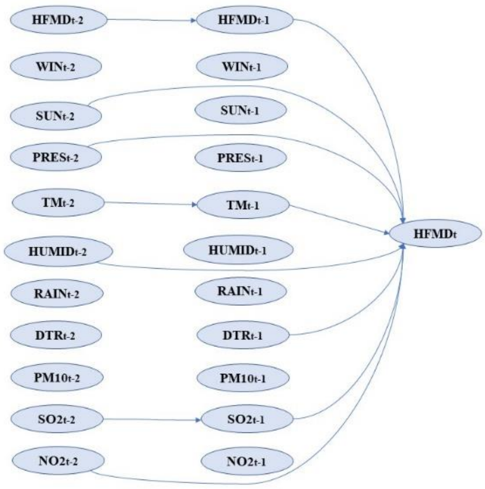

DBN_③

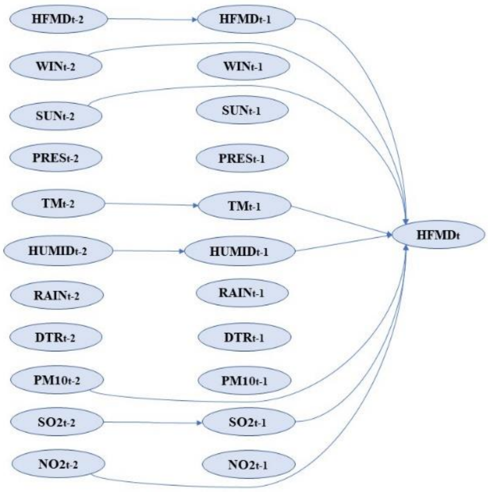

DBN_④

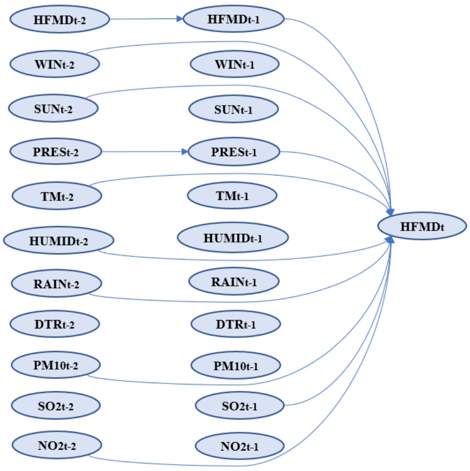

DBN_⑤

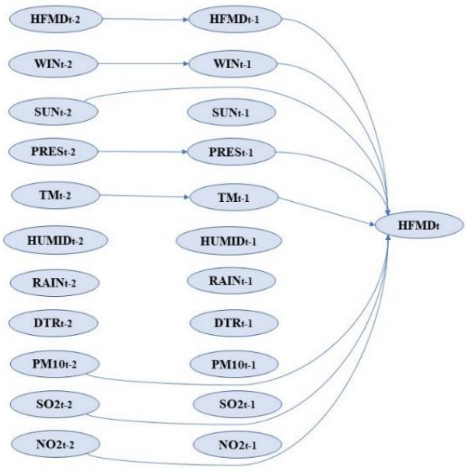

DBN_⑥

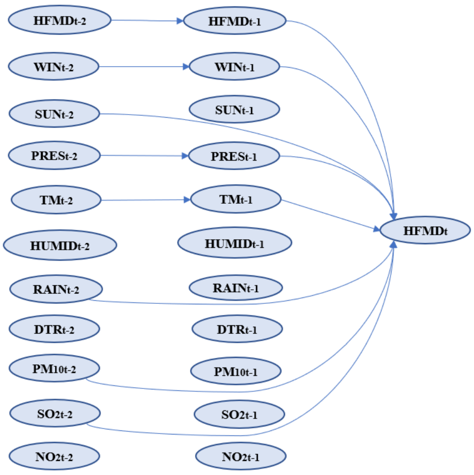

DBN_⑦

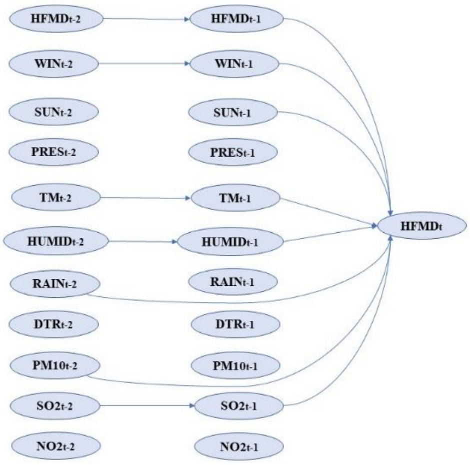

DBN_⑧

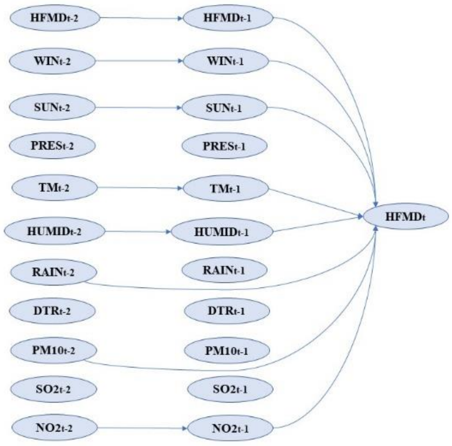

DBN_⑨

Fig. S1. Correlation patterns of the DBN_①~⑨ models

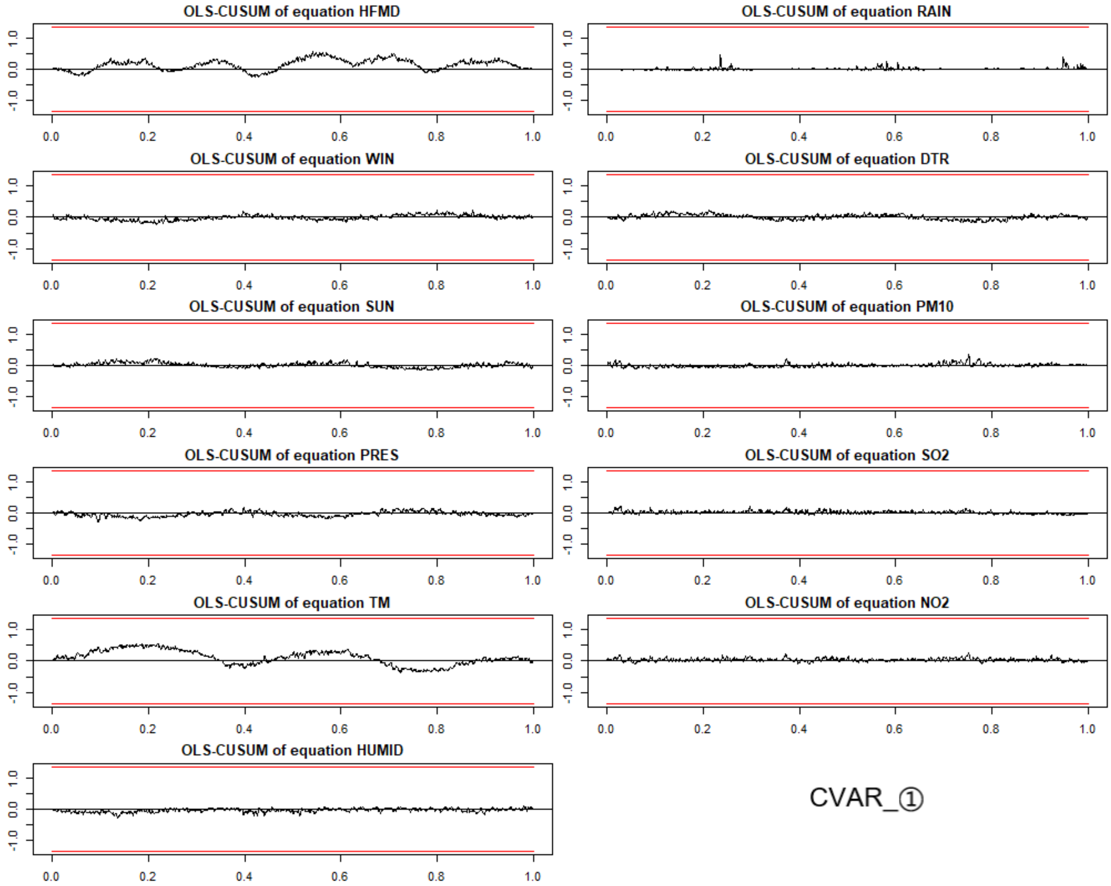
OLS-CUSUM of equation HFMD
OLS-CUSUM of equation RAIN
OLS-CUSUM of equation WIN
OLS-CUSUM of equation DTR
OLS-CUSUM of equation SUN
OLS-CUSUM of equation PM10
OLS-CUSUM of equation PRES
OLS-CUSUM of equation SO2
OLS-CUSUM of equation TM
OLS-CUSUM of equation NO2
OLS-CUSUM of equation HUMID

CVAR_①

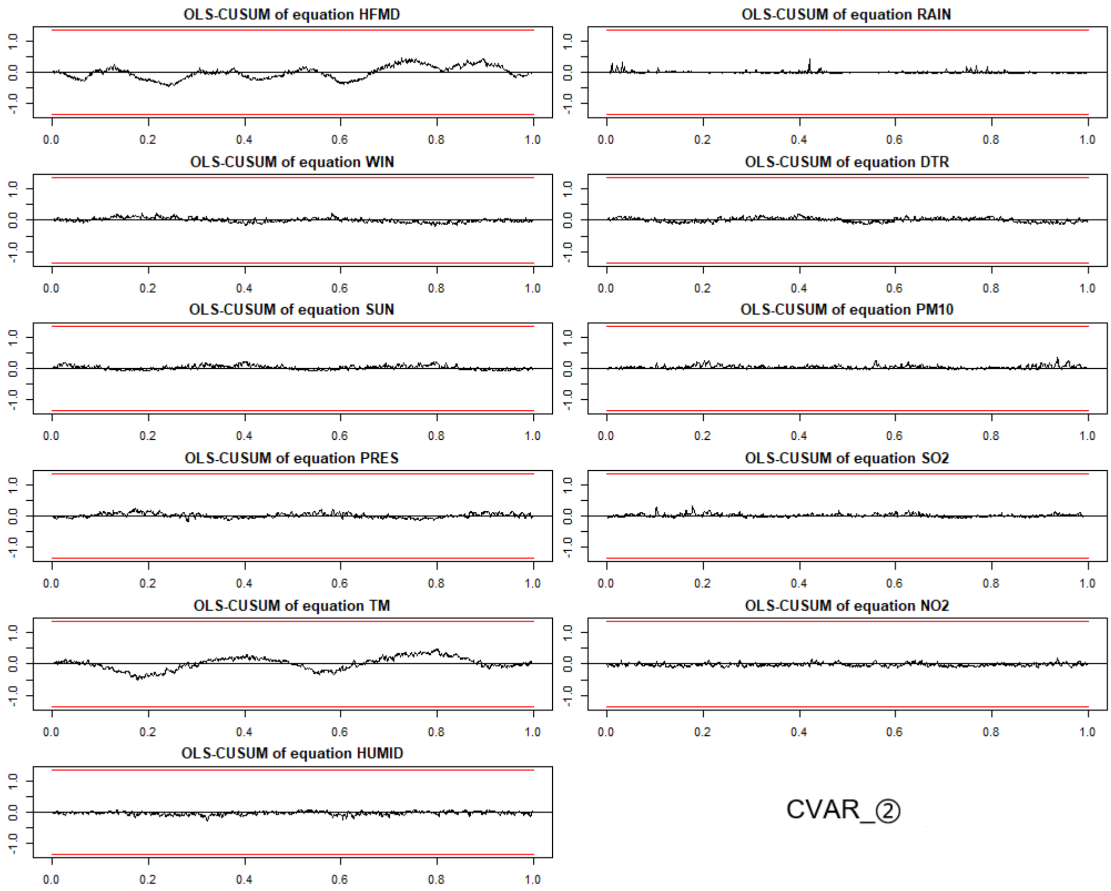
OLS-CUSUM of equation HFMD
OLS-CUSUM of equation RAIN
OLS-CUSUM of equation WIN
OLS-CUSUM of equation DTR
OLS-CUSUM of equation SUN
OLS-CUSUM of equation PM10
OLS-CUSUM of equation PRES
OLS-CUSUM of equation SO2
OLS-CUSUM of equation TM
OLS-CUSUM of equation NO2
OLS-CUSUM of equation HUMID
CVAR_②

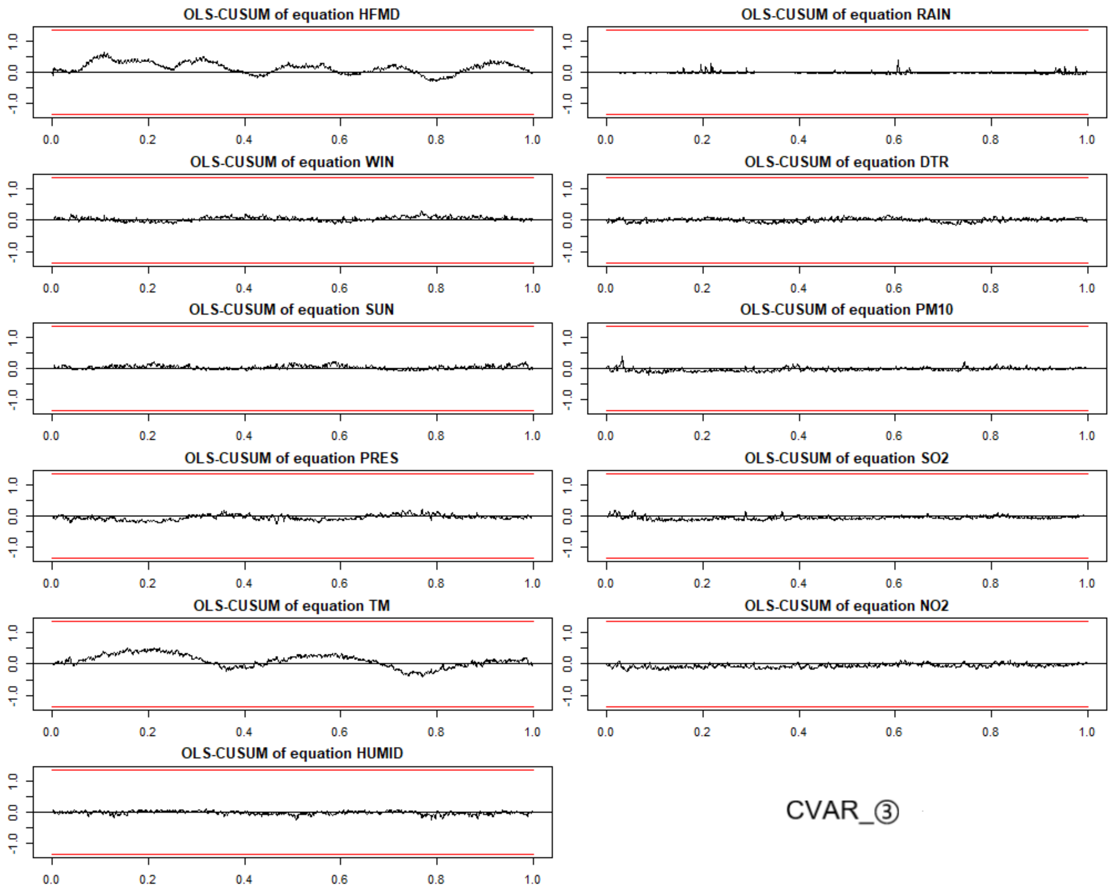
OLS-CUSUM of equation HFMD
OLS-CUSUM of equation RAIN
OLS-CUSUM of equation WIN
OLS-CUSUM of equation DTR
OLS-CUSUM of equation SUN
OLS-CUSUM of equation PM10
OLS-CUSUM of equation PRES
OLS-CUSUM of equation SO2
OLS-CUSUM of equation TM
OLS-CUSUM of equation NO2
OLS-CUSUM of equation HUMID
CVAR_③

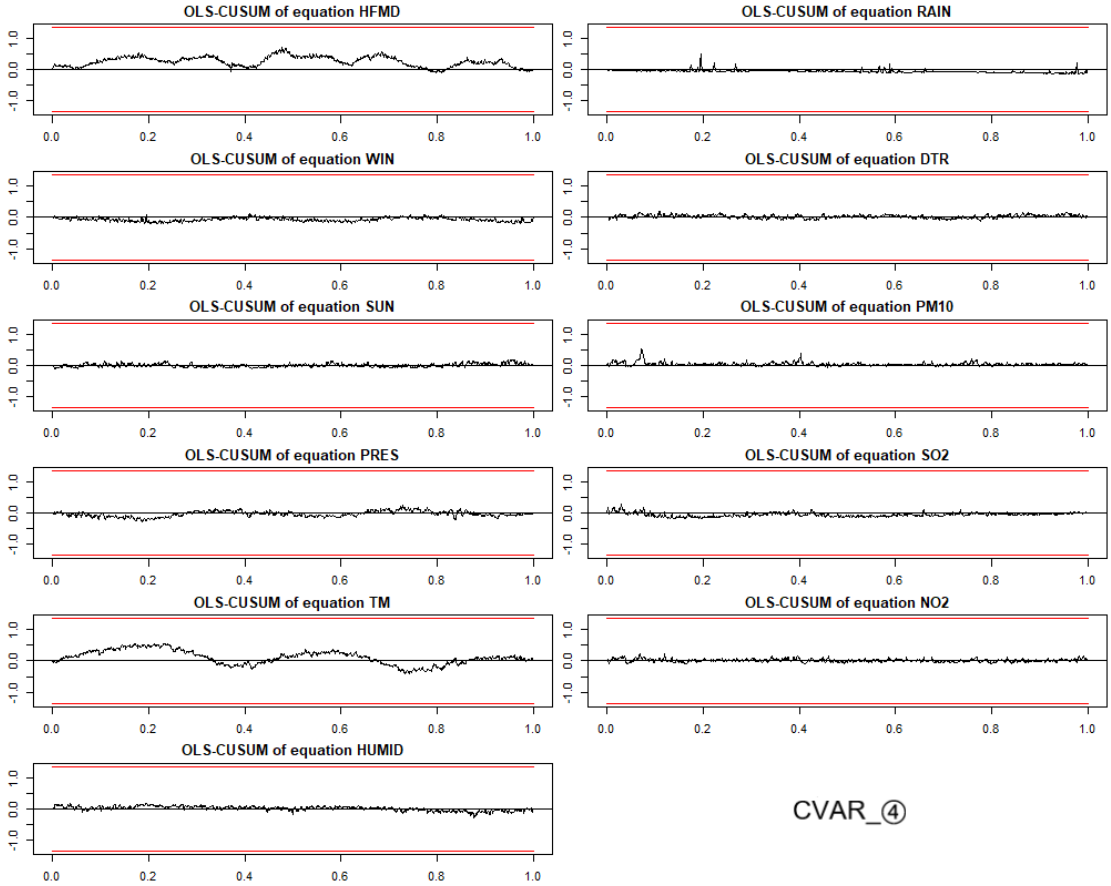
OLS-CUSUM of equation HFMD
OLS-CUSUM of equation RAIN
OLS-CUSUM of equation WIN
OLS-CUSUM of equation DTR
OLS-CUSUM of equation SUN
OLS-CUSUM of equation PM10
OLS-CUSUM of equation PRES
OLS-CUSUM of equation SO2
OLS-CUSUM of equation TM
OLS-CUSUM of equation NO2
OLS-CUSUM of equation HUMID

CVAR_④

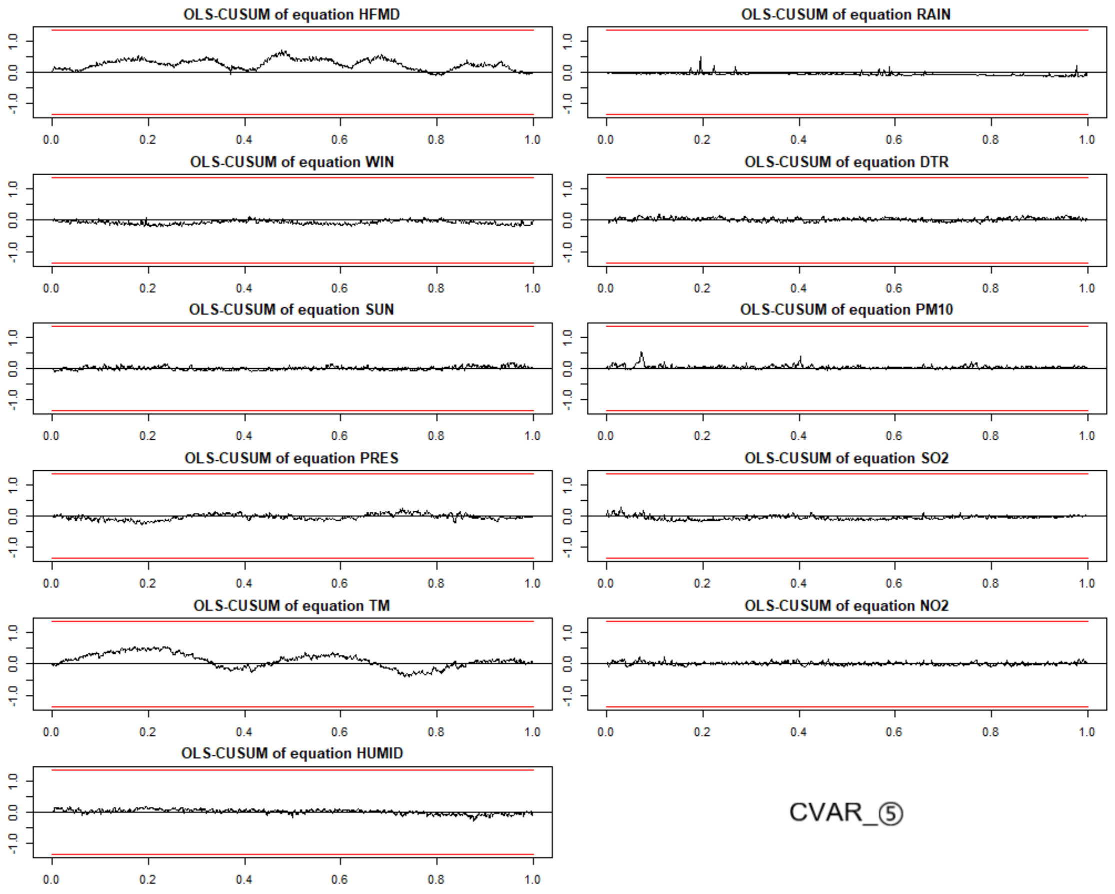
OLS-CUSUM of equation HFMD
OLS-CUSUM of equation RAIN
OLS-CUSUM of equation WIN
OLS-CUSUM of equation DTR
OLS-CUSUM of equation SUN
OLS-CUSUM of equation PM10
OLS-CUSUM of equation PRES
OLS-CUSUM of equation SO2
OLS-CUSUM of equation TM
OLS-CUSUM of equation NO2
OLS-CUSUM of equation HUMID
-1.0 0.0 1.0
0.0 0.2 0.4 0.6 0.8 1.0
CVAR_⑤

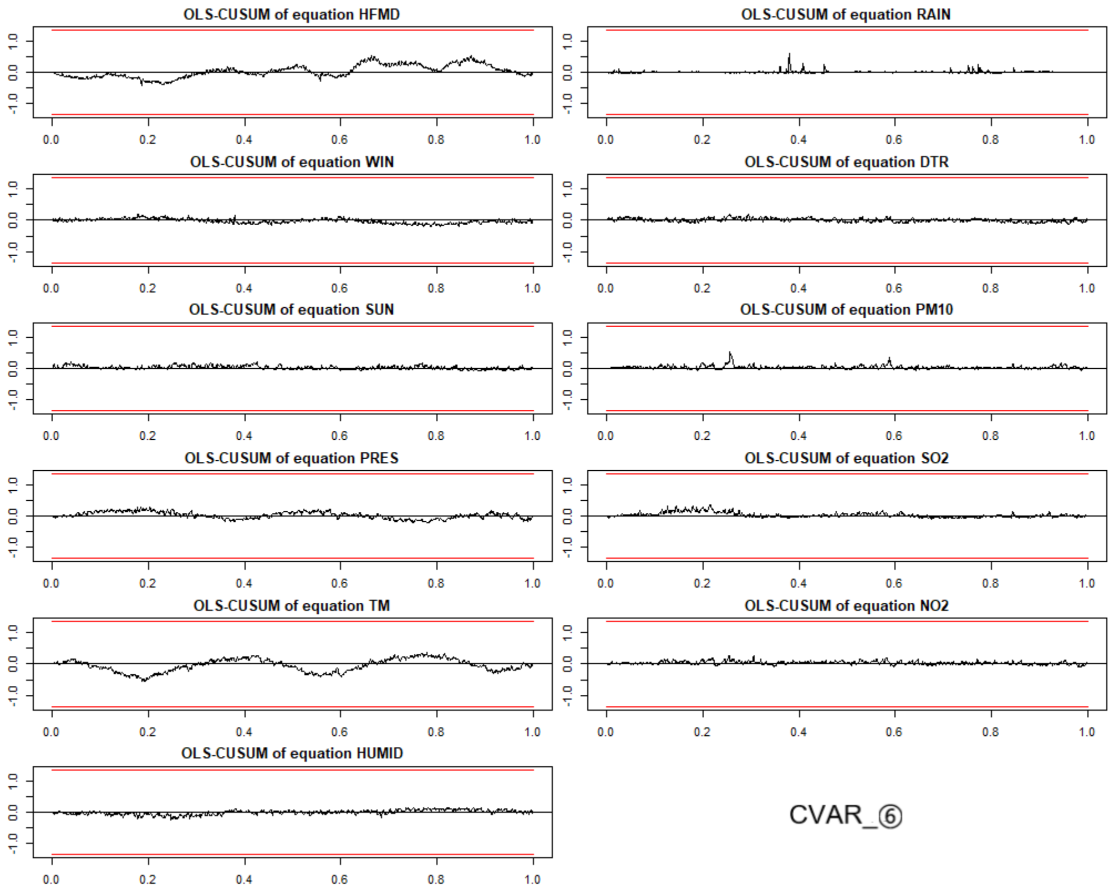
OLS-CUSUM of equation HFMD
OLS-CUSUM of equation RAIN
OLS-CUSUM of equation WIN
OLS-CUSUM of equation DTR
OLS-CUSUM of equation SUN
OLS-CUSUM of equation PM10
OLS-CUSUM of equation PRES
OLS-CUSUM of equation SO2
OLS-CUSUM of equation TM
OLS-CUSUM of equation NO2
OLS-CUSUM of equation HUMID
-1.0
0.0
1.0
0.0
0.2
0.4
0.6
0.8
1.0

CVAR_⑥

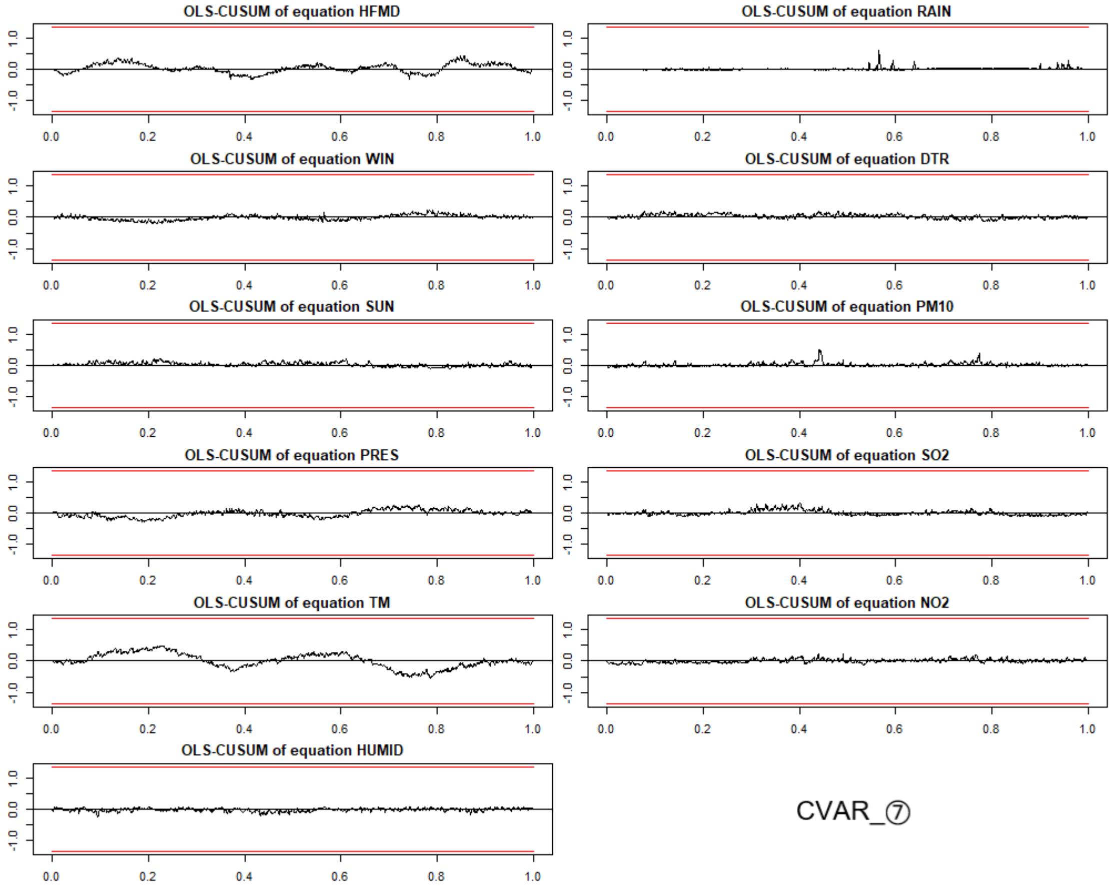
OLS-CUSUM of equation HFMD
OLS-CUSUM of equation RAIN
OLS-CUSUM of equation WIN
OLS-CUSUM of equation DTR
OLS-CUSUM of equation SUN
OLS-CUSUM of equation PM10
OLS-CUSUM of equation PRES
OLS-CUSUM of equation SO2
OLS-CUSUM of equation TM
OLS-CUSUM of equation NO2
OLS-CUSUM of equation HUMID

CVAR_⑦

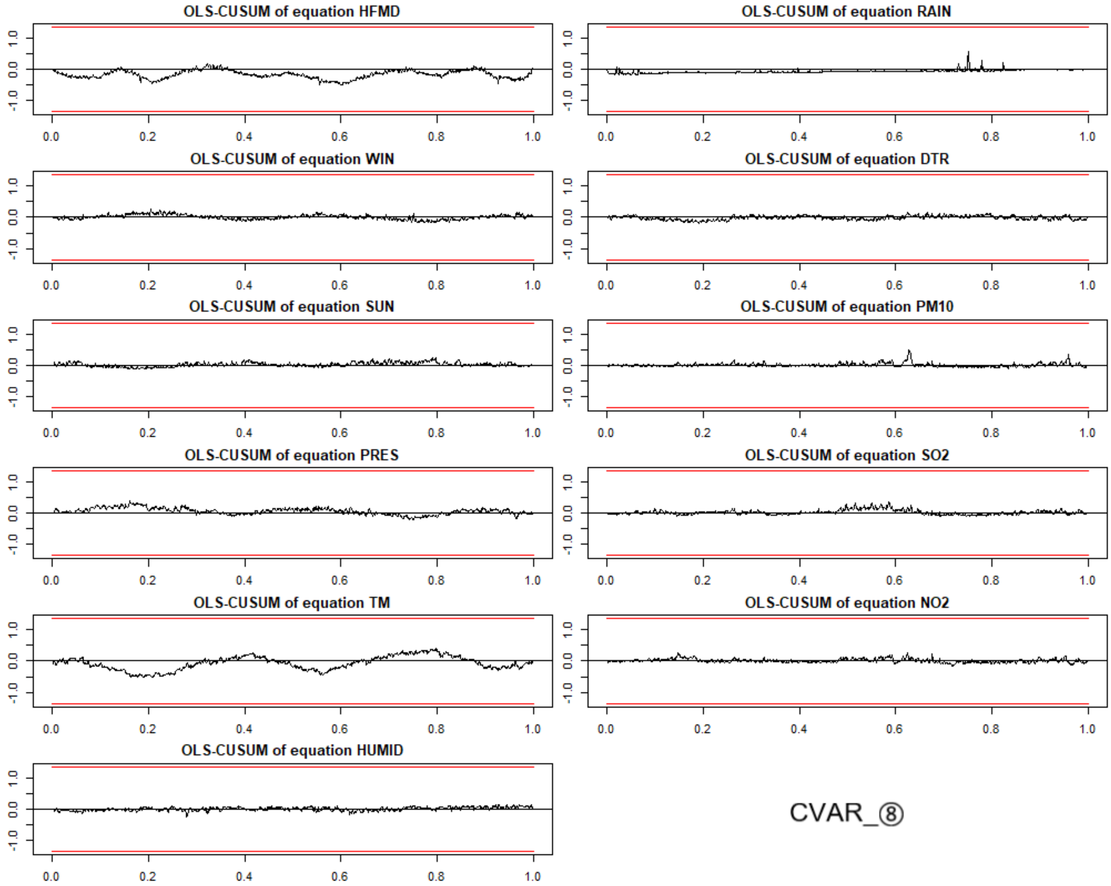
OLS-CUSUM of equation HFMD
OLS-CUSUM of equation RAIN
OLS-CUSUM of equation WIN
OLS-CUSUM of equation DTR
OLS-CUSUM of equation SUN
OLS-CUSUM of equation PM10
OLS-CUSUM of equation PRES
OLS-CUSUM of equation SO2
OLS-CUSUM of equation TM
OLS-CUSUM of equation NO2
OLS-CUSUM of equation HUMID
CVAR_⑧

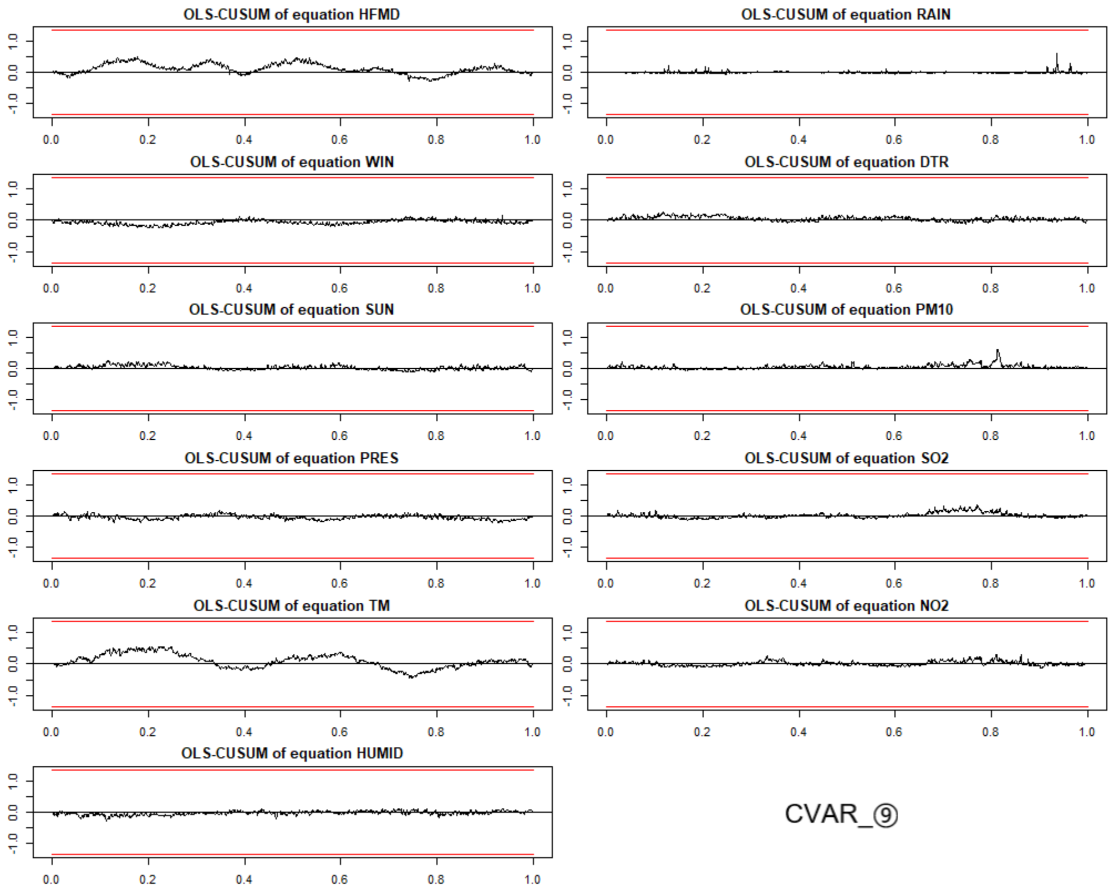

Fig. S2. The OLS-CUSUM results of the CVAR_①~⑨ models

Table S7. The averaged RMSE and MAPE of the dynamic prediction and direct prediction of CVAR_①~⑨ models

Model	RMSE					MAPE(%)				
	2 days ahead	3 days ahead	7 days ahead	10 days ahead	Direct prediction	2 days ahead	3 days ahead	7 days ahead	10 days ahead	Direct prediction
①	1.110	1.112	1.111	1.112	1.119	100.34	102.84	109.82	112.23	115.68
②	1.185	1.202	1.391	1.471	1.987	99.73	103.11	109.41	112.31	115.91
③	1.188	1.199	1.481	1.552	1.664	101.07	101.24	106.62	110.91	115.99
④	1.181	1.190	1.173	1.126	1.201	98.89	102.92	107.32	110.93	118.13
⑤	1.167	1.176	1.149	1.127	1.046	99.75	102.97	106.82	109.33	117.35
⑥	1.164	1.164	1.139	1.091	1.633	98.24	103.31	106.21	109.44	118.02
⑦	1.160	1.160	1.144	1.170	1.559	101.53	103.99	107.78	109.58	117.80
⑧	1.162	1.163	1.117	1.096	1.370	101.39	103.88	107.70	109.54	117.70
⑨	1.158	1.153	1.105	1.581	0.993	102.41	103.04	107.24	109.41	117.57
Average	1.164	1.169	1.201	1.258	1.397	100.37	103.03	107.66	110.41	117.13

Table S8. The averaged RMSE and MAPE of the dynamic prediction and direct prediction of ARIMAX_①~⑨ models

Model	RMSE					MAPE(%)				
	2 days ahead	3 days ahead	7 days ahead	10 days ahead	Direct prediction	2 days ahead	3 days ahead	7 days ahead	10 days ahead	Direct prediction
①	1.282	1.281	1.298	1.312	1.413	113.31	114.02	119.67	127.32	137.34
②	1.904	1.938	1.917	2.005	2.293	140.60	146.39	150.32	142.59	139.24
③	1.727	1.720	1.798	1.776	1.946	140.21	148.12	148.46	141.23	159.21
④	1.481	1.490	1.493	1.426	1.581	123.57	128.49	137.31	137.06	155.09
⑤	1.467	1.476	1.499	1.497	1.512	120.72	123.25	124.08	122.73	126.69
⑥	1.592	1.646	1.660	1.685	1.917	125.27	126.93	133.38	148.89	128.20
⑦	1.664	1.696	1.743	1.743	1.856	115.70	108.52	113.24	112.96	133.01
⑧	1.389	1.393	1.492	1.501	1.655	115.17	112.30	104.86	108.25	105.74
⑨	1.388	1.383	1.505	1.681	1.693	105.47	110.79	114.09	125.92	143.05
Average	1.544	1.558	1.601	1.625	1.763	122.22	124.31	127.27	129.66	136.40

VAR_①~⑨ equations:

VAR_①:

$$\begin{pmatrix} HFMD_t \\ WIN_t \\ SUN_t \\ PRES_t \\ TM_t \\ HUMID_t \\ RAIN_t \\ DTR_t \\ PM10_t \\ SO2_t \\ NO2_t \end{pmatrix} = \begin{pmatrix} 0.0058 \\ -0.003 \\ 0.0169 \\ 0.0028 \\ 0.0215 \\ -0.017 \\ -0.007 \\ 0.0207 \\ -0.040 \\ -0.042 \\ -0.006 \end{pmatrix} +$$

$$\begin{pmatrix} -0.195 & -0.103 & 0.0045 & -0.003 & -0.062 & -0.001 & 0.0018 & 0.0103 & 0.0006 & 0.0015 & -0.006 \\ 0.0019 & -0.552 & -0.010 & -0.007 & 0.0651 & 0.0024 & 0.0024 & -0.005 & 0.0014 & 0.0010 & -0.001 \\ 0.2192 & 0.1233 & -0.559 & 0.1104 & -0.127 & -0.019 & -0.002 & 0.0136 & -0.003 & 0.0039 & 0.0015 \\ -0.279 & 1.0320 & -0.076 & 0.0583 & -0.077 & -0.018 & -0.018 & -0.082 & -0.003 & 0.0083 & -0.005 \\ 0.1462 & -0.290 & -0.006 & -0.054 & -0.016 & 0.0487 & -0.004 & 0.1250 & 0.0035 & 0.0008 & -0.005 \\ -0.281 & -0.129 & -0.060 & -0.123 & 0.1188 & -0.329 & 0.0134 & -0.119 & -0.011 & -0.023 & 0.0154 \\ -0.402 & 2.0606 & -0.092 & -0.343 & 0.3829 & -0.014 & -0.431 & -0.131 & -0.001 & -0.027 & 0.0643 \\ 0.1794 & -0.007 & 0.1067 & 0.0661 & -0.224 & -0.023 & -0.004 & -0.456 & -0.008 & 0.0097 & 0.0170 \\ 0.7254 & -22.74 & -0.723 & -1.512 & 1.1799 & -0.338 & -0.073 & 3.2115 & -0.291 & -0.536 & 0.3346 \\ 0.3484 & -2.976 & -0.130 & -0.567 & 0.1684 & -0.030 & -0.001 & 0.4484 & 0.0086 & -0.389 & 0.0794 \\ 0.3093 & -4.174 & 0.0692 & 0.0924 & 0.3711 & 0.1183 & -0.009 & 0.9073 & -0.015 & -0.034 & -0.232 \end{pmatrix} \begin{pmatrix} HFMD_{t-1} \\ WIN_{t-1} \\ SUN_{t-1} \\ PRES_{t-1} \\ TM_{t-1} \\ HUMID_{t-1} \\ RAIN_{t-1} \\ DTR_{t-1} \\ PM10_{t-1} \\ SO2_{t-1} \\ NO2_{t-1} \end{pmatrix} +$$

$$
\begin{pmatrix}
-0.145 & -0.116 & 0.0323 & -0.010 & -0.101 & -0.012 & -0.002 & -0.017 & 0.0005 & -0.002 & -0.001 \\
-0.025 & -0.261 & -0.011 & -0.006 & 0.0739 & 0.0064 & 0.0009 & -0.002 & 0.0009 & 0.0007 & -0.001 \\
0.0139 & 0.1558 & -0.219 & 0.0588 & -0.201 & 0.0040 & -0.006 & -0.023 & -0.002 & -0.021 & 0.0062 \\
-0.058 & -0.053 & 0.0130 & -0.305 & -0.334 & -0.036 & -0.004 & -0.109 & -0.005 & 0.0143 & -0.008 \\
0.0598 & 0.0228 & -0.020 & -0.016 & -0.146 & 0.0148 & 0.0018 & 0.0601 & 0.0024 & -0.006 & 0.0102 \\
-0.268 & -0.301 & -0.057 & 0.3251 & 0.5978 & -0.202 & 0.0020 & -0.094 & -0.004 & 0.0001 & -0.028 \\
-0.435 & 1.1564 & -0.077 & -0.055 & 0.2243 & 0.0167 & -0.332 & -0.107 & 0.0010 & 0.0767 & -0.057 \\
0.0179 & -0.074 & 0.1856 & -0.002 & -0.282 & 0.0084 & -0.012 & -0.241 & -0.003 & -0.019 & -0.001 \\
-1.598 & -7.992 & -0.225 & -0.644 & -1.265 & -0.016 & 0.1284 & 2.6626 & -0.236 & 0.0151 & 0.5896 \\
0.0189 & -0.581 & -0.066 & 0.1694 & 0.3016 & 0.0217 & 0.0544 & 0.3928 & -0.005 & -0.320 & 0.1805 \\
0.2820 & -0.837 & -0.023 & 0.0265 & -0.014 & -0.006 & 0.0284 & 0.6853 & -0.007 & -0.086 & -0.073
\end{pmatrix}
\begin{pmatrix}
HFMD_{t-2} \\ WIN_{t-2} \\ SUN_{t-2} \\ PRES_{t-2} \\ TM_{t-2} \\ HUMID_{t-2} \\ RAIN_{t-2} \\ DTR_{t-2} \\ PM10_{t-2} \\ SO2_{t-2} \\ NO2_{t-2}
\end{pmatrix}
$$

VAR_②:

$$
\begin{pmatrix}
HFMD_{t} \\ WIN_{t} \\ SUN_{t} \\ PRES_{t} \\ TM_{t} \\ HUMID_{t} \\ RAIN_{t} \\ DTR_{t} \\ PM10_{t} \\ SO2_{t} \\ NO2_{t}
\end{pmatrix}
=
\begin{pmatrix}
0.0001 \\ 0.0022 \\ -0.007 \\ 0.0261 \\ -0.016 \\ -0.011 \\ -0.044 \\ -0.003 \\ 0.0860 \\ -0.004 \\ 0.0412
\end{pmatrix}
+
$$

$$
\begin{pmatrix}
-0.219 & -0.121 & 0.0085 & -0.015 & -0.087 & -0.007 & 0.0019 & 0.0019 & 0.0007 & -0.002 & -0.004 \\
-0.012 & -0.562 & -0.007 & -0.015 & 0.0592 & 0.0031 & 0.0026 & -0.001 & 0.0010 & 0.0027 & -0.001 \\
0.1106 & 0.0687 & -0.555 & 0.0951 & -0.197 & -0.022 & -0.004 & 0.0125 & -0.004 & 0.0064 & 0.0094 \\
-0.333 & 0.9717 & -0.109 & 0.0232 & 0.0446 & 0.0034 & -0.002 & -0.030 & -0.004 & 0.0107 & -0.012 \\
0.1601 & -0.278 & -0.010 & -0.051 & -0.015 & 0.0479 & -0.001 & 0.1159 & 0.0024 & 0.0035 & -0.002 \\
-0.260 & -0.075 & -0.088 & -0.087 & 0.1629 & -0.320 & 0.0051 & -0.089 & -0.004 & -0.064 & 0.0209 \\
-0.628 & 2.1183 & -0.241 & -0.349 & 0.2826 & 0.0074 & -0.432 & 0.0780 & -0.004 & -0.026 & 0.0494 \\
0.2660 & 0.1247 & 0.1447 & 0.0865 & -0.237 & -0.020 & -0.006 & -0.467 & -0.008 & 0.0174 & 0.0207 \\
1.4138 & -19.82 & -0.414 & -0.908 & 0.2178 & -0.421 & -0.014 & 3.1962 & -0.261 & -0.638 & 0.5624 \\
0.6792 & -1.808 & 0.0580 & -0.411 & -0.275 & -0.102 & 0.0015 & 0.4161 & 0.0085 & -0.432 & 0.1433 \\
0.9587 & -2.892 & 0.2239 & 0.0842 & -0.209 & 0.0468 & -0.014 & 0.9337 & -0.011 & -0.073 & -0.184
\end{pmatrix}
\begin{pmatrix}
HFMD_{t-1} \\ WIN_{t-1} \\ SUN_{t-1} \\ PRES_{t-1} \\ TM_{t-1} \\ HUMID_{t-1} \\ RAIN_{t-1} \\ DTR_{t-1} \\ PM10_{t-1} \\ SO2_{t-1} \\ NO2_{t-1}
\end{pmatrix}
+
$$

$$\begin{pmatrix} -0.149 & -0.108 & 0.0335 & -0.012 & -0.105 & -0.008 & -0.002 & -0.013 & 0.0004 & -0.003 & -0.002 \\ -0.036 & -0.231 & -0.007 & -0.009 & 0.0798 & 0.0050 & -0.001 & -0.001 & 0.0004 & 0.0010 & 0.0002 \\ 0.0001 & 0.0835 & -0.222 & 0.0136 & -0.109 & 0.0030 & -0.010 & -0.041 & -0.002 & -0.034 & 0.0056 \\ -0.098 & -0.137 & -0.020 & -0.332 & -0.265 & -0.037 & -0.002 & -0.096 & -0.005 & 0.0122 & -0.016 \\ 0.0892 & 0.0101 & 0.0076 & -0.015 & -0.104 & 0.0172 & 0.0020 & 0.0327 & 0.0029 & -0.009 & 0.0119 \\ -0.381 & -0.031 & -0.073 & 0.3606 & 0.2876 & -0.209 & 0.0026 & -0.010 & -0.003 & -0.011 & -0.008 \\ -0.317 & 1.6025 & -0.165 & 0.0088 & 0.0769 & -0.001 & -0.346 & -0.047 & -0.002 & 0.0543 & -0.038 \\ 0.0045 & -0.111 & 0.1833 & -0.021 & -0.240 & -0.001 & -0.013 & -0.272 & -0.003 & -0.030 & 0.0046 \\ -1.557 & -4.905 & 0.1280 & -0.115 & -1.570 & 0.0219 & 0.1069 & 2.4385 & -0.220 & 0.0973 & 0.6096 \\ 0.1509 & 0.1623 & 0.0557 & 0.1709 & 0.3170 & 0.0240 & 0.0495 & 0.2899 & -0.004 & -0.319 & 0.1779 \\ 0.5880 & -0.172 & 0.1269 & 0.0289 & -0.323 & -0.031 & 0.0049 & 0.6314 & -0.009 & -0.115 & -0.031 \end{pmatrix} \begin{pmatrix} HFMD_{t-2} \\ WIN_{t-2} \\ SUN_{t-2} \\ PRES_{t-2} \\ TM_{t-2} \\ HUMID_{t-2} \\ RAIN_{t-2} \\ DTR_{t-2} \\ PM10_{t-2} \\ SO2_{t-2} \\ NO2_{t-2} \end{pmatrix}$$

VAR_③:

$$\begin{pmatrix} HFMD_t \\ WIN_t \\ SUN_t \\ PRES_t \\ TM_t \\ HUMID_t \\ RAIN_t \\ DTR_t \\ PM10_t \\ SO2_t \\ NO2_t \end{pmatrix} = \begin{pmatrix} 0.0048 \\ -0.002 \\ 0.0123 \\ -0.023 \\ 0.0218 \\ 0.6369 \\ -0.016 \\ 0.0136 \\ -0.066 \\ -0.293 \\ -0.025 \end{pmatrix} +$$

$$\begin{pmatrix} -0.230 & -0.033 & 0.0102 & 0.0062 & -0.043 & -0.007 & 0.0022 & -0.003 & 0.0005 & -0.005 & 0.0004 \\ 0.0167 & -0.545 & -0.011 & -0.009 & 0.0691 & 0.0052 & 0.0028 & -0.001 & 0.0011 & 0.0026 & 0.0002 \\ 0.0947 & 0.2427 & -0.548 & 0.0779 & -0.193 & -0.021 & -0.010 & -0.001 & -0.005 & -0.003 & 0.0183 \\ -0.245 & 1.0256 & -0.124 & 0.0366 & 0.1674 & 0.0139 & -0.005 & -0.005 & -0.004 & 0.0138 & -0.019 \\ 0.0724 & -0.246 & -0.005 & -0.063 & -0.067 & 0.0378 & 0.0027 & 0.1046 & 0.0022 & -0.001 & 0.0012 \\ -0.169 & -0.311 & -0.127 & -0.079 & 0.0406 & -0.326 & -0.001 & -0.024 & -0.003 & -0.053 & -0.001 \\ -0.389 & 1.4388 & -0.157 & -0.216 & 0.4578 & 0.0384 & -0.440 & 0.0164 & -0.003 & -0.009 & 0.0308 \\ 0.1321 & 0.2799 & 0.1655 & 0.0442 & -0.274 & -0.019 & -0.005 & -0.483 & -0.008 & 0.0075 & 0.0292 \\ 1.2200 & -20.30 & 0.1529 & -0.988 & -1.630 & -0.489 & 0.1475 & 2.9758 & -0.266 & -0.562 & 0.4825 \\ 0.6546 & -1.725 & 0.0967 & -0.471 & -0.657 & -0.122 & 0.0253 & 0.4291 & 0.0120 & -0.466 & 0.1436 \\ 0.4056 & -2.885 & 0.1419 & -0.064 & -0.576 & 0.0219 & 0.0045 & 1.0797 & -0.014 & -0.071 & -0.224 \end{pmatrix} \begin{pmatrix} HFMD_{t-1} \\ WIN_{t-1} \\ SUN_{t-1} \\ PRES_{t-1} \\ TM_{t-1} \\ HUMID_{t-1} \\ RAIN_{t-1} \\ DTR_{t-1} \\ PM10_{t-1} \\ SO2_{t-1} \\ NO2_{t-1} \end{pmatrix} +$$

$$\begin{pmatrix} -0.072 & -0.118 & 0.0428 & -0.003 & -0.131 & -0.019 & -0.001 & -0.018 & 0.0005 & -0.002 & -0.004 \\ -0.022 & -0.209 & -0.003 & -0.010 & 0.0747 & 0.0049 & 0.0007 & -0.007 & 0.0003 & 0.0003 & 0.0021 \\ 0.0869 & 0.1828 & -0.229 & 0.0138 & -0.094 & -0.004 & -0.008 & -0.029 & -0.004 & -0.037 & 0.0118 \\ -0.205 & -0.136 & -0.011 & -0.334 & -0.229 & -0.050 & -0.002 & -0.117 & -0.005 & 0.0022 & -0.012 \\ 0.0845 & 0.0572 & 0.0016 & -0.019 & -0.134 & 0.0154 & 0.0032 & 0.0355 & 0.0025 & -0.008 & 0.0076 \\ -0.117 & -0.416 & -0.094 & 0.3926 & 0.3586 & -0.191 & 0.0066 & 0.0642 & -0.001 & -0.003 & -0.001 \\ -0.322 & 0.6383 & 0.1255 & -0.017 & 0.2613 & -0.001 & -0.362 & -0.209 & -0.004 & 0.0484 & 0.0074 \\ 0.0889 & -0.016 & 0.1819 & -0.033 & -0.278 & -0.007 & -0.010 & -0.265 & -0.004 & -0.032 & 0.0040 \\ 0.1069 & -4.638 & 0.2327 & -0.060 & -1.798 & 0.0795 & 0.1477 & 3.2405 & -0.192 & 0.1450 & 0.3957 \\ 0.5398 & 0.4862 & -0.098 & 0.2240 & 0.3950 & 0.0577 & 0.0450 & 0.5068 & 0.0002 & -0.293 & 0.1171 \\ 0.9886 & -0.134 & -0.010 & 0.0393 & -0.639 & -0.022 & -0.002 & 0.8361 & -0.003 & -0.099 & -0.087 \end{pmatrix} \begin{pmatrix} HFMD_{t-2} \\ WIN_{t-2} \\ SUN_{t-2} \\ PRES_{t-2} \\ TM_{t-2} \\ HUMID_{t-2} \\ RAIN_{t-2} \\ DTR_{t-2} \\ PM10_{t-2} \\ SO2_{t-2} \\ NO2_{t-2} \end{pmatrix}$$

VAR_④:

$$\begin{pmatrix} HFMD_t \\ WIN_t \\ SUN_t \\ PRES_t \\ TM_t \\ HUMID_t \\ RAIN_t \\ DTR_t \\ PM10_t \\ SO2_t \\ NO2_t \end{pmatrix} = \begin{pmatrix} -0.007 \\ 0.0033 \\ 0.0020 \\ 0.0165 \\ -0.015 \\ -0.020 \\ 0.0042 \\ 0.0066 \\ 0.0412 \\ -0.010 \\ 0.0148 \end{pmatrix} +$$

$$\begin{pmatrix} -0.169 & 0.0377 & 0.0165 & 0.0213 & -0.026 & 0.0007 & -0.001 & -0.234 & 0.0001 & -0.011 & 0.0024 \\ -0.011 & -0.580 & -0.013 & -0.016 & 0.0497 & 10.002 & 0.0030 & 0.0021 & 0.0009 & 0.0031 & -0.002 \\ 0.2170 & 0.3231 & -0.550 & 0.0669 & -0.126 & -0.018 & -0.012 & -0.057 & -0.006 & -0.005 & 0.0240 \\ -0.183 & 1.0780 & -0.111 & 0.0621 & 0.2464 & 0.0038 & -0.000 & -0.064 & -0.004 & 0.0125 & -0.003 \\ 0.0615 & -0.293 & -0.006 & -0.072 & -0.099 & 0.0410 & 0.0011 & 0.0976 & 0.0017 & 0.0027 & 0.0001 \\ -0.187 & -0.448 & -0.108 & -0.117 & -0.176 & -0.384 & 0.0011 & 0.0087 & -0.001 & -0.066 & -0.001 \\ -0.566 & 1.0216 & -0.039 & -0.191 & 0.2690 & 0.0344 & -0.446 & -0.044 & -0.001 & 0.0065 & 0.0128 \\ 0.0101 & 0.3501 & 0.3607 & 0.2067 & 0.0271 & -0.218 & -0.007 & -0.008 & -0.568 & -0.009 & 0.0095 \\ 2.4298 & -18.01 & 0.2892 & -1.262 & -4.337 & -0.640 & 0.0757 & 2.9559 & -0.270 & -0.540 & 0.5333 \\ 0.9183 & -1.559 & 0.1099 & -0.407 & -0.661 & -0.091 & 0.0195 & 0.4924 & 0.0085 & -0.465 & 0.1303 \\ 0.7882 & -2.181 & 0.3975 & -0.234 & -0.978 & -0.022 & -0.009 & 0.8803 & -0.018 & -0.075 & -0.183 \end{pmatrix} \begin{pmatrix} HFMD_{t-1} \\ WIN_{t-1} \\ SUN_{t-1} \\ PRES_{t-1} \\ TM_{t-1} \\ HUMID_{t-1} \\ RAIN_{t-1} \\ DTR_{t-1} \\ PM10_{t-1} \\ SO2_{t-1} \\ NO2_{t-1} \end{pmatrix} +$$

$$
\begin{pmatrix}
-0.121 & -0.127 & 0.0333 & -0.007 & -0.123 & -0.016 & -0.001 & -0.024 & -0.001 & -0.004 & -0.007 \\
-0.026 & -0.252 & -0.010 & -0.011 & 0.0665 & 20.002 & 0.0010 & 0.0016 & 0.0003 & -0.000 & 0.0024 \\
0.0431 & 0.2428 & -0.190 & 0.0292 & -0.030 & 0.0046 & -0.006 & -0.068 & -0.005 & -0.034 & 0.0120 \\
-0.104 & -0.154 & 0.0205 & -0.327 & -0.303 & -0.062 & 0.0022 & -0.168 & -0.005 & -0.002 & -0.006 \\
0.0658 & 0.1074 & 0.0149 & -0.004 & -0.080 & 0.0282 & 0.0032 & 0.0325 & 0.0019 & -0.011 & 0.0087 \\
-0.131 & -0.641 & -0.196 & 0.2739 & -0.024 & -0.257 & -0.001 & 0.1440 & 0.0079 & -0.001 & -0.017 \\
-0.312 & 0.5828 & 0.1360 & -0.076 & 0.3345 & 0.0176 & -0.358 & -0.134 & -0.001 & 0.0410 & 0.0237 \\
0.0345 & 0.0657 & 0.0895 & 0.2261 & -0.011 & -0.129 & 0.0066 & -0.339 & -0.006 & -0.025 & 0.0110 \\
-0.201 & -4.727 & 0.5397 & -0.423 & -2.565 & 0.0696 & 0.0579 & 2.5045 & -0.183 & 0.2382 & 0.3443 \\
0.5621 & 0.2823 & -0.132 & 0.1145 & 0.3397 & 0.0329 & 0.0369 & 0.4456 & -0.001 & -0.276 & 0.1106 \\
0.9107 & 0.0773 & 0.1702 & -0.061 & -0.964 & -0.024 & -0.025 & 0.6361 & -0.004 & -0.077 & -0.083
\end{pmatrix}
\begin{pmatrix}
HFMD_{t-2} \\ WIN_{t-2} \\ SUN_{t-2} \\ PRES_{t-2} \\ TM_{t-2} \\ HUMID_{t-2} \\ RAIN_{t-2} \\ DTR_{t-2} \\ PM10_{t-2} \\ SO2_{t-2} \\ NO2_{t-2}
\end{pmatrix}
$$

VAR_⑤:

$$
\begin{pmatrix}
HFMD_t \\ WIN_t \\ SUN_t \\ PRES_t \\ TM_t \\ HUMID_t \\ RAIN_t \\ DTR_t \\ PM10_t \\ SO2_t \\ NO2_t
\end{pmatrix}
=
\begin{pmatrix}
0.0069 \\ -0.001 \\ -0.008 \\ -0.012 \\ 0.0220 \\ 0.0452 \\ 0.0139 \\ -0.003 \\ -0.181 \\ -0.238 \\ -0.010
\end{pmatrix}
+
$$

$$
\begin{pmatrix}
-0.254 & 0.0300 & 0.0214 & 0.0188 & -0.045 & -0.004 & -0.001 & -0.023 & 0.0006 & -0.007 & 0.0024 \\
0.0312 & -0.582 & -0.021 & -0.009 & 0.0409 & 10.001 & 0.0113 & 0.0188 & 0.0011 & 0.0028 & -0.002 \\
0.1058 & 0.1151 & -0.515 & 0.0143 & -0.188 & -0.012 & -0.015 & -0.050 & -0.011 & -0.020 & 0.0230 \\
-0.175 & 1.0141 & -0.120 & 0.0910 & 0.1746 & 0.0011 & -0.011 & -0.050 & -0.007 & 0.0095 & -0.003 \\
0.0200 & -0.333 & 0.0278 & -0.066 & -0.090 & 0.0457 & 0.0035 & 0.0804 & 0.0016 & 0.0014 & 0.0020 \\
-0.076 & -0.448 & -0.171 & -0.067 & -0.013 & -0.405 & 0.0121 & -0.007 & 0.0051 & -0.030 & -0.016 \\
-0.373 & 0.7084 & -0.083 & -0.182 & 0.1369 & 0.0486 & -0.460 & 0.0103 & -0.001 & 0.0573 & -0.011 \\
0.1332 & 0.1340 & 0.2339 & -0.035 & -0.379 & -0.021 & -0.011 & -0.590 & -0.008 & -0.006 & 0.0407 \\
2.5305 & -14.33 & 1.1823 & -1.280 & -4.165 & -0.564 & 0.1235 & 2.4251 & -0.261 & -0.479 & 0.6245 \\
0.5333 & -0.985 & 0.3691 & -0.234 & -0.611 & -0.019 & 0.0260 & 0.3933 & 0.0084 & -0.450 & 0.1443 \\
0.0990 & -1.085 & 0.6959 & -0.219 & -0.921 & -0.005 & 0.0051 & 0.6839 & -0.021 & -0.041 & -0.141
\end{pmatrix}
\begin{pmatrix}
HFMD_{t-1} \\ WIN_{t-1} \\ SUN_{t-1} \\ PRES_{t-1} \\ TM_{t-1} \\ HUMID_{t-1} \\ RAIN_{t-1} \\ DTR_{t-1} \\ PM10_{t-1} \\ SO2_{t-1} \\ NO2_{t-1}
\end{pmatrix}
+
$$

$$\begin{pmatrix} -0.104 & -0.157 & 0.0347 & -0.004 & -0.133 & -0.022 & -0.001 & -0.025 & -0.001 & -0.002 & -0.003 \\ -0.326 & -0.230 & -0.008 & -0.012 & 0.0655 & 20.003 & 0.0010 & 0.0046 & 0.0002 & 0.0013 & 0.0009 \\ 0.0329 & 0.1551 & -0.189 & 0.0275 & -0.078 & 0.0225 & -0.010 & -0.067 & -0.005 & -0.024 & 0.0126 \\ -0.118 & -0.096 & 0.0318 & -0.310 & -0.392 & -0.069 & 0.0010 & -0.149 & -0.005 & 0.0034 & 0.0058 \\ 0.0589 & 0.0643 & 0.0251 & -0.021 & -0.118 & 0.0319 & 0.0022 & 0.0318 & 0.0022 & -0.008 & 0.0072 \\ 0.1710 & -0.565 & -0.228 & 0.3259 & 0.1500 & -0.256 & 0.0078 & 0.1025 & 0.0089 & -0.009 & -0.023 \\ -0.267 & 0.0674 & 0.1309 & -0.155 & 0.4330 & 0.0329 & -0.366 & -0.026 & -0.003 & 0.0690 & -0.017 \\ 0.0531 & -0.031 & 0.2307 & -0.006 & -0.148 & 0.0146 & -0.009 & -0.366 & -0.005 & -0.029 & 0.0161 \\ 1.1972 & -1.791 & 0.4688 & 0.0730 & -2.143 & 0.0481 & 0.0425 & 2.1396 & -0.179 & 0.3304 & 0.2678 \\ 0.4579 & 0.4230 & -0.057 & 0.0955 & 0.3324 & 20.063 & 0.0271 & 0.4012 & 0.0001 & -0.238 & 0.0652 \\ 0.8971 & 0.1299 & 0.3036 & 0.0025 & -0.988 & -0.006 & -0.037 & 0.4794 & -0.002 & -0.066 & -0.142 \end{pmatrix} \begin{pmatrix} HFMD_{t-2} \\ WIN_{t-2} \\ SUN_{t-2} \\ PRES_{t-2} \\ TM_{t-2} \\ HUMID_{t-2} \\ RAIN_{t-2} \\ DTR_{t-2} \\ PM10_{t-2} \\ SO2_{t-2} \\ NO2_{t-2} \end{pmatrix}$$

VAR_⑥:

$$\begin{pmatrix} HFMD_t \\ WIN_t \\ SUN_t \\ PRES_t \\ TM_t \\ HUMID_t \\ RAIN_t \\ DTR_t \\ PM10_t \\ SO2_t \\ NO2_t \end{pmatrix} = \begin{pmatrix} -0.011 \\ 0.0002 \\ -0.003 \\ 0.0261 \\ -0.015 \\ -0.029 \\ 0.0045 \\ -0.002 \\ -0.023 \\ -0.007 \\ -0.013 \end{pmatrix} +$$

$$\begin{pmatrix} -0.263 & 0.0604 & 0.0180 & 0.0096 & -0.037 & -0.002 & -0.001 & -0.017 & 0.0011 & -0.006 & -0.001 \\ -0.039 & -0.490 & -0.016 & -0.009 & 0.0474 & 10.004 & 0.0015 & 0.0107 & 0.0003 & 0.0030 & -0.001 \\ 0.0690 & 0.0421 & -0.517 & 0.0156 & -0.220 & 0.0042 & -0.015 & -0.027 & -0.008 & -0.013 & 0.0195 \\ -0.172 & 1.1891 & -0.093 & 0.1818 & 0.2451 & 0.0240 & -0.014 & -0.078 & -0.006 & -0.003 & 0.0006 \\ 0.0030 & -0.371 & 0.0322 & -0.105 & -0.110 & 0.0477 & 0.0032 & 0.0769 & -0.001 & 0.0030 & 0.0040 \\ -0.105 & -1.251 & -0.113 & -0.036 & -0.242 & -0.467 & 0.0239 & -0.078 & 0.0181 & 0.0136 & -0.053 \\ -0.388 & 0.5132 & -0.082 & -0.156 & -0.009 & 10.016 & -0.470 & -0.001 & -0.001 & 0.1503 & -0.024 \\ 0.1109 & 0.1861 & 0.2544 & -0.030 & -0.364 & -0.005 & -0.012 & -0.606 & -0.007 & -0.012 & 0.0414 \\ 2.7010 & -12.04 & 1.1016 & -0.149 & -2.718 & -0.610 & 0.1660 & 1.9222 & -0.290 & -0.353 & 0.8043 \\ 0.5456 & -0.875 & 0.3170 & -0.157 & -0.594 & -0.073 & 0.0173 & 0.2024 & 0.0015 & -0.475 & 0.1488 \\ 0.8409 & -2.121 & 0.7106 & -0.234 & -1.310 & -0.087 & 0.0185 & 0.4542 & -0.016 & -0.010 & -0.174 \end{pmatrix} \begin{pmatrix} HFMD_{t-1} \\ WIN_{t-1} \\ SUN_{t-1} \\ PRES_{t-1} \\ TM_{t-1} \\ HUMID_{t-1} \\ RAIN_{t-1} \\ DTR_{t-1} \\ PM10_{t-1} \\ SO2_{t-1} \\ NO2_{t-1} \end{pmatrix} +$$

$$\begin{pmatrix} -0.123 & -0.061 & 0.0396 & 0.0024 & -0.100 & -0.007 & -0.002 & -0.022 & -0.001 & -0.004 & -0.001 \\ -0.030 & -0.213 & -0.006 & -0.004 & 0.0636 & 20.003 & 0.0006 & 0.0014 & 0.0002 & 0.0033 & 0.0005 \\ 0.0136 & 0.1174 & -0.209 & 0.0225 & -0.074 & 0.0209 & -0.012 & -0.038 & -0.004 & -0.033 & 0.0076 \\ -0.112 & 0.1770 & 0.0837 & -0.268 & -0.364 & -0.051 & -0.005 & -0.168 & -0.006 & -0.008 & 0.0114 \\ 0.0475 & 0.0212 & 0.0338 & -0.032 & -0.081 & 0.0366 & 0.0016 & 0.0287 & 0.0026 & -0.010 & 0.0033 \\ 0.2200 & -0.980 & -0.191 & 0.3030 & 0.1507 & -0.256 & 0.0230 & -0.016 & 0.0095 & 0.0495 & -0.017 \\ -0.255 & -0.082 & 0.0992 & -0.117 & 0.4675 & 20.045 & -0.360 & -0.005 & -0.004 & 0.1514 & -0.008 \\ 0.0397 & -0.011 & 0.1724 & 0.0107 & -0.115 & 0.0075 & -0.010 & -0.345 & -0.003 & -0.039 & 0.0137 \\ 1.8079 & 0.7435 & -0.558 & 0.7339 & -1.002 & -0.020 & 0.0083 & 2.0167 & -0.061 & -0.171 & 0.1736 \\ 0.5036 & 0.2683 & -0.253 & 0.0361 & 0.2663 & -0.035 & 0.0156 & 0.3448 & 0.0138 & -0.257 & 0.0206 \\ 0.9793 & 0.3181 & 0.2033 & -0.020 & -0.856 & -0.002 & -0.045 & 0.4362 & 0.0084 & -0.066 & -0.163 \end{pmatrix} \begin{pmatrix} HFMD_{t-2} \\ WIN_{t-2} \\ SUN_{t-2} \\ PRES_{t-2} \\ TM_{t-2} \\ HUMID_{t-2} \\ RAIN_{t-2} \\ DTR_{t-2} \\ PM10_{t-2} \\ SO2_{t-2} \\ NO2_{t-2} \end{pmatrix}$$

VAR_⑦:

$$\begin{pmatrix} HFMD_t \\ WIN_t \\ SUN_t \\ PRES_t \\ TM_t \\ HUMID_t \\ RAIN_t \\ DTR_t \\ PM10_t \\ SO2_t \\ NO2_t \end{pmatrix} = \begin{pmatrix} 0.0065 \\ -0.007 \\ 0.0066 \\ -0.012 \\ 0.0216 \\ 0.0060 \\ -0.004 \\ 0.0048 \\ -0.133 \\ -0.019 \\ -0.045 \end{pmatrix} +$$

$$\begin{pmatrix} -0.246 & 0.1015 & 0.0161 & 0.0143 & -0.027 & -0.003 & -0.001 & -0.012 & 0.0012 & -0.004 & -0.001 \\ -0.008 & -0.536 & -0.020 & -0.006 & 0.0482 & 10.006 & 0.0011 & 0.0103 & 0.0002 & 0.0020 & 0.0011 \\ 0.2064 & -0.089 & -0.466 & 0.0708 & -0.231 & 0.0160 & -0.026 & -0.015 & -0.007 & -0.004 & 0.0213 \\ -0.229 & 1.1802 & -0.076 & 0.1658 & 0.1534 & 0.0278 & -0.018 & -0.079 & -0.011 & -0.010 & 0.0059 \\ 0.0284 & -0.362 & 0.0352 & -0.100 & -0.113 & 0.0420 & 0.0081 & 0.0746 & -0.000 & 0.0045 & 0.0074 \\ -0.336 & -1.421 & -0.199 & -0.028 & 0.0775 & -0.441 & 0.0205 & -0.022 & 0.0170 & 0.0325 & -0.080 \\ -0.186 & -0.041 & -0.095 & -0.064 & -0.085 & 0.0357 & -0.626 & 0.0541 & -0.002 & 0.0865 & -0.044 \\ 0.2525 & -0.073 & 0.2921 & -0.024 & -0.409 & -0.007 & -0.012 & -0.659 & -0.012 & -0.023 & 0.0393 \\ 2.7734 & -15.09 & 1.1056 & -0.217 & -3.135 & -0.824 & 0.1974 & 1.1565 & -0.308 & -0.385 & 0.8110 \\ 0.6438 & -0.826 & 0.2525 & -0.095 & -0.456 & -0.093 & 0.0405 & 0.1570 & -0.011 & -0.510 & 0.1470 \\ 0.6993 & -2.626 & 0.7390 & -0.250 & -1.301 & -0.127 & 0.0123 & 0.3382 & -0.022 & 0.0318 & -0.149 \end{pmatrix} \begin{pmatrix} HFMD_{t-1} \\ WIN_{t-1} \\ SUN_{t-1} \\ PRES_{t-1} \\ TM_{t-1} \\ HUMID_{t-1} \\ RAIN_{t-1} \\ DTR_{t-1} \\ PM10_{t-1} \\ SO2_{t-1} \\ NO2_{t-1} \end{pmatrix} +$$

$$\begin{pmatrix} -0.106 & -0.069 & 0.0217 & 0.0100 & -0.100 & -0.006 & -0.001 & -0.008 & -0.001 & -0.005 & -0.002 \\ -0.041 & -0.274 & -0.005 & 0.0003 & 0.0677 & 20.004 & 0.0030 & 0.0039 & 0.0006 & -0.002 & 0.0018 \\ 0.1101 & -0.173 & -0.192 & -0.020 & -0.192 & 0.0268 & -0.020 & -0.044 & -0.005 & -0.003 & 0.0064 \\ -0.108 & 0.0627 & 0.0699 & -0.278 & -0.347 & -0.043 & -0.006 & -0.149 & -0.005 & -0.024 & 0.0121 \\ 0.0945 & 0.0252 & 0.0287 & -0.024 & -0.091 & 0.0394 & 0.0022 & 0.0365 & 0.0016 & 0.0003 & 0.0026 \\ -0.026 & -1.094 & -0.261 & 0.2794 & 0.2687 & -0.273 & 0.0234 & -0.006 & 0.0101 & 0.0615 & -0.034 \\ -0.224 & -1.006 & 0.1083 & -0.126 & 0.6033 & 0.0620 & -0.364 & 0.0653 & 0.0006 & 0.0314 & 0.0346 \\ 0.1874 & -0.343 & 0.2014 & -0.052 & -0.249 & 0.0052 & -0.013 & -0.379 & -0.004 & -0.015 & 0.0121 \\ 2.4447 & -3.461 & 0.2153 & 0.2962 & -1.608 & -0.149 & 0.0546 & 1.0223 & -0.092 & -0.153 & 0.2189 \\ 0.5496 & 0.1973 & -0.111 & 0.0105 & 0.0379 & -0.059 & 0.0111 & 0.1768 & 0.0068 & -0.254 & 0.0342 \\ 1.1175 & -0.272 & 0.3809 & -0.144 & -1.015 & -0.035 & -0.046 & 0.2014 & -0.002 & -0.017 & -0.155 \end{pmatrix} \begin{pmatrix} HFMD_{t-2} \\ WIN_{t-2} \\ SUN_{t-2} \\ PRES_{t-2} \\ TM_{t-2} \\ HUMID_{t-2} \\ RAIN_{t-2} \\ DTR_{t-2} \\ PM10_{t-2} \\ SO2_{t-2} \\ NO2_{t-2} \end{pmatrix}$$

VAR_⑧:

$$\begin{pmatrix} HFMD_{t} \\ WIN_{t} \\ SUN_{t} \\ PRES_{t} \\ TM_{t} \\ HUMID_{t} \\ RAIN_{t} \\ DTR_{t} \\ PM10_{t} \\ SO2_{t} \\ NO2_{t} \end{pmatrix} = \begin{pmatrix} -0.011 \\ 0.0019 \\ -0.002 \\ 0.0017 \\ -0.015 \\ -0.000 \\ 0.0061 \\ -0.004 \\ 0.0197 \\ 0.0035 \\ -0.017 \end{pmatrix} +$$

$$\begin{pmatrix} -0.271 & 0.1103 & 0.0402 & -0.000 & -0.090 & -0.012 & -0.004 & -0.030 & 0.0009 & -0.002 & -0.001 \\ -0.016 & -0.486 & -0.011 & -0.009 & 0.0434 & 0.0051 & 0.0033 & 0.0095 & 0.0006 & 0.0021 & -0.010 \\ 0.3184 & -0.121 & -0.451 & 0.0526 & -0.276 & 10.011 & -0.032 & -0.092 & -0.005 & 0.0075 & 0.0169 \\ -0.158 & 1.2034 & -0.059 & 0.1805 & 0.0973 & 0.0377 & -0.022 & -0.110 & -0.014 & 0.0017 & 0.0122 \\ 0.0354 & -0.363 & 0.0256 & -0.120 & -0.160 & 0.0402 & 0.0099 & 0.0988 & 0.0013 & 0.0080 & 0.0101 \\ -0.450 & -1.134 & -0.111 & 0.0387 & 0.1950 & -0.446 & 0.0261 & -0.074 & 0.0260 & 0.0038 & -0.065 \\ -0.211 & -0.079 & -0.020 & -0.017 & -0.164 & 0.0239 & -0.517 & -0.025 & 0.0047 & 0.1130 & -0.044 \\ 0.3230 & -0.350 & 0.2986 & 0.0135 & -0.371 & 0.0087 & -0.011 & -0.685 & -0.005 & -0.023 & 0.0365 \\ 2.4199 & -14.66 & 1.2452 & -0.244 & -2.751 & -0.324 & 0.2054 & 1.1542 & -0.282 & -0.608 & 0.9614 \\ 0.4840 & -0.912 & 0.1753 & -0.044 & -0.175 & -0.093 & 0.0324 & 0.1335 & -0.006 & -0.510 & 0.1142 \\ 0.5951 & -3.433 & 0.7832 & -0.114 & -0.821 & -0.105 & 0.0161 & 0.2301 & -0.024 & -0.003 & -0.154 \end{pmatrix} \begin{pmatrix} HFMD_{t-1} \\ WIN_{t-1} \\ SUN_{t-1} \\ PRES_{t-1} \\ TM_{t-1} \\ HUMID_{t-1} \\ RAIN_{t-1} \\ DTR_{t-1} \\ PM10_{t-1} \\ SO2_{t-1} \\ NO2_{t-1} \end{pmatrix} +$$

$$\begin{pmatrix} -0.158 & -0.168 & 0.0190 & -0.020 & -0.146 & -0.015 & -0.003 & -0.017 & -0.003 & -0.003 & 0.0021 \\ -0.040 & -0.300 & -0.006 & -0.007 & 0.0593 & 0.0032 & 0.0024 & 0.0049 & 0.0001 & -0.001 & 0.0013 \\ 0.1321 & -0.275 & -0.225 & -0.008 & -0.245 & 20.036 & -0.021 & -0.027 & -0.001 & -0.021 & 0.0043 \\ 0.0381 & 0.1586 & 0.0341 & -0.312 & -0.366 & -0.018 & -0.010 & -0.135 & -0.009 & -0.017 & 0.0298 \\ 0.0910 & 0.1023 & -0.003 & -0.016 & -0.064 & 0.0422 & 0.0028 & 0.0694 & 0.0022 & -0.000 & 0.0007 \\ -0.213 & -0.718 & -0.120 & 0.2109 & 0.2537 & -0.295 & 0.0213 & -0.202 & 0.0073 & 0.0809 & -0.041 \\ -0.262 & -0.912 & 0.1676 & -0.188 & 0.5287 & 0.0569 & -0.362 & 0.0403 & -0.005 & 0.0610 & 0.0514 \\ 0.2126 & -0.651 & 0.1858 & -0.000 & -0.266 & 0.0272 & -0.011 & -0.361 & 0.0010 & -0.032 & 0.0146 \\ 2.0729 & -1.011 & -0.025 & 0.9211 & 0.3171 & 0.0937 & 0.0701 & 0.9237 & -0.066 & -0.198 & 0.2026 \\ 0.3992 & -0.280 & -0.151 & 0.0452 & 0.1936 & -0.066 & 0.0117 & 0.1608 & 0.0134 & -0.276 & 0.0022 \\ 0.7428 & -0.391 & 0.2605 & 0.0250 & -0.553 & -0.009 & -0.044 & 0.2377 & 0.0127 & -0.076 & -0.188 \end{pmatrix} \begin{pmatrix} HFMD_{t-2} \\ WIN_{t-2} \\ SUN_{t-2} \\ PRES_{t-2} \\ TM_{t-2} \\ HUMID_{t-2} \\ RAIN_{t-2} \\ DTR_{t-2} \\ PM10_{t-2} \\ SO2_{t-2} \\ NO2_{t-2} \end{pmatrix}$$

VAR_⑨:

$$\begin{pmatrix} HFMD_t \\ WIN_t \\ SUN_t \\ PRES_t \\ TM_t \\ HUMID_t \\ RAIN_t \\ DTR_t \\ PM10_t \\ SO2_t \\ NO2_t \end{pmatrix} = \begin{pmatrix} 0.0007 \\ -0.003 \\ 0.0226 \\ -0.011 \\ 0.0247 \\ -0.021 \\ -0.006 \\ 0.0196 \\ -0.126 \\ 0.0066 \\ -0.014 \end{pmatrix} +$$

$$\begin{pmatrix} -0.279 & 0.0471 & 0.0102 & -0.009 & -0.078 & -0.014 & -0.005 & -0.008 & 0.0010 & -0.003 & -0.007 \\ -0.023 & -0.548 & -0.015 & -0.004 & 0.0528 & 0.0062 & -0.001 & 0.0153 & 0.0003 & -0.011 & -0.001 \\ 0.2024 & -0.239 & -0.425 & 0.0795 & -0.198 & 0.0206 & -0.018 & -0.088 & -0.002 & 0.0083 & -0.011 \\ -0.091 & 1.1313 & -0.109 & 0.2080 & 0.0922 & 0.0384 & -0.112 & -0.041 & -0.018 & 0.0216 & -0.000 \\ 0.0302 & -0.400 & 0.0379 & -0.122 & -0.194 & 0.0371 & 0.0108 & 0.0968 & 0.0011 & 0.0051 & 0.0083 \\ -0.339 & -0.908 & -0.129 & 0.0149 & 0.2508 & -0.455 & 0.0060 & -0.150 & 0.0273 & 0.0256 & -0.043 \\ -0.204 & 1.1490 & -0.211 & 0.0377 & -0.048 & 0.0303 & -0.658 & 0.1152 & -0.001 & 0.1176 & -0.021 \\ 0.2841 & -0.278 & 0.2109 & 0.0088 & -0.410 & 0.0035 & -0.006 & -0.585 & -0.003 & -0.007 & 0.0140 \\ 1.5945 & -13.45 & 2.0299 & 0.0102 & -2.625 & -0.630 & 0.0815 & 0.2908 & -0.263 & -0.356 & 0.7863 \\ 0.4337 & -1.089 & 0.2839 & -0.078 & -0.199 & -0.105 & 0.0344 & -0.022 & -0.008 & -0.480 & 0.1066 \\ 0.7245 & -3.540 & 1.0895 & -0.094 & -0.874 & -0.110 & 0.0200 & -0.024 & -0.022 & 0.0282 & -0.162 \end{pmatrix} \begin{pmatrix} HFMD_{t-1} \\ WIN_{t-1} \\ SUN_{t-1} \\ PRES_{t-1} \\ TM_{t-1} \\ HUMID_{t-1} \\ RAIN_{t-1} \\ DTR_{t-1} \\ PM10_{t-1} \\ SO2_{t-1} \\ NO2_{t-1} \end{pmatrix} +$$

$$\begin{pmatrix} -0.157 & -0.176 & 0.0222 & -0.026 & -0.169 & -0.020 & -0.006 & -0.025 & -0.002 & 0.0061 & -0.002 \\ -0.047 & -0.301 & -0.010 & -0.012 & 0.0619 & 0.0040 & 0.0026 & 0.0071 & 0.0004 & -0.001 & -0.001 \\ 0.1328 & -0.348 & -0.180 & 0.0365 & -0.267 & 0.0402 & -0.016 & -0.032 & -0.001 & 0.0260 & -0.002 \\ 0.0045 & 0.2379 & 0.0274 & -0.247 & -0.267 & 0.0077 & -0.011 & -0.052 & -0.008 & -0.013 & 0.0179 \\ 0.0935 & 0.0862 & -0.012 & -0.030 & -0.056 & 0.0420 & 0.0042 & 0.0932 & 0.0011 & 0.0081 & 0.0012 \\ -0.265 & -0.605 & -0.120 & 0.2068 & 0.3592 & -0.285 & 0.0066 & -0.284 & 0.0079 & 0.0538 & -0.042 \\ -0.348 & 0.0274 & 0.0067 & -0.119 & 0.4511 & 0.0576 & -0.356 & 0.0671 & -0.001 & 0.0739 & -0.004 \\ 0.2718 & -0.532 & 0.1608 & 0.0229 & -0.347 & 0.0218 & -0.012 & -0.326 & 0.0007 & 0.0185 & 0.0134 \\ 1.9548 & -0.477 & 0.7018 & 0.9154 & 0.0110 & 0.0856 & 0.0143 & 0.5426 & -0.054 & -0.302 & 0.2369 \\ 0.4023 & -0.173 & 0.0518 & 0.0971 & -0.034 & -0.074 & 0.0024 & 0.0074 & 0.0144 & -0.324 & 0.0282 \\ 1.0230 & -0.563 & 0.4231 & 0.0917 & -0.370 & 0.0231 & -0.031 & 0.1371 & 0.0112 & -0.081 & -0.187 \end{pmatrix} \begin{pmatrix} HFMD_{t-2} \\ WIN_{t-2} \\ SUN_{t-2} \\ PRES_{t-2} \\ TM_{t-2} \\ HUMID_{t-2} \\ RAIN_{t-2} \\ DTR_{t-2} \\ PM10_{t-2} \\ SO2_{t-2} \\ NO2_{t-2} \end{pmatrix}$$

CVAR_①~⑨ equations:

CVAR_①:

$$\begin{pmatrix} HFMD_t \\ WIN_t \\ SUN_t \\ PRES_t \\ TM_t \\ HUMID_t \\ RAIN_t \\ DTR_t \\ PM10_t \\ SO2_t \\ NO2_t \end{pmatrix} = \begin{pmatrix} 0.0074 \\ -0.002 \\ 0.0155 \\ 0.0032 \\ 0.0213 \\ -0.021 \\ -0.002 \\ 0.0180 \\ -0.067 \\ -0.039 \\ 0.0002 \end{pmatrix} +$$

$$
\begin{pmatrix}
-0.197 & -0.081 & 0.0021 & 0.0000 & -0.053 & 0.0000 & 0.0000 & 0.0156 & 0.0000 & 0.0000 & -0.005 \\
0.0000 & -0.554 & -0.010 & -0.006 & 0.0635 & 0.0024 & 0.0025 & -0.006 & 0.0014 & 0.0010 & -0.001 \\
0.0000 & 0.1221 & -0.562 & 0.1086 & -0.119 & -0.018 & -0.003 & 0.0179 & -0.003 & 0.0043 & 0.0008 \\
0.0000 & 1.0265 & -0.071 & 0.0606 & -0.087 & -0.019 & 0.0025 & -0.087 & -0.003 & 0.0080 & -0.004 \\
0.0000 & -0.287 & -0.008 & -0.056 & -0.005 & 0.0492 & -0.004 & 0.1282 & 0.0034 & 0.0010 & -0.005 \\
0.0000 & -0.156 & -0.056 & -0.115 & 0.0935 & -0.331 & 0.0143 & -0.128 & -0.010 & -0.024 & 0.0162 \\
0.0000 & 2.0273 & -0.089 & -0.327 & 0.3241 & -0.016 & -0.429 & -0.141 & -0.001 & -0.028 & 0.0648 \\
0.0000 & -0.012 & 0.1030 & 0.0629 & -0.210 & -0.022 & -0.004 & -0.451 & -0.008 & 0.0101 & 0.0162 \\
0.0000 & -22.94 & -0.757 & -1.500 & 1.1466 & -0.336 & -0.073 & 3.2147 & -0.292 & -0.536 & 0.3294 \\
0.0000 & -2.975 & -0.136 & -0.570 & 0.1840 & -0.028 & -0.002 & 0.4561 & 0.0082 & -0.388 & 0.0781 \\
0.0000 & -4.146 & 0.0661 & 0.0861 & 0.3941 & 0.1197 & -0.011 & 0.9168 & -0.015 & -0.033 & -0.233
\end{pmatrix}
\begin{pmatrix}
HFMD_{t-1} \\ WIN_{t-1} \\ SUN_{t-1} \\ PRES_{t-1} \\ TM_{t-1} \\ HUMID_{t-1} \\ RAIN_{t-1} \\ DTR_{t-1} \\ PM10_{t-1} \\ SO2_{t-1} \\ NO2_{t-1}
\end{pmatrix}
+
$$

$$
\begin{pmatrix}
-0.108 & -0.088 & 0.0191 & 0.0000 & -0.093 & -0.012 & -0.004 & 0.0000 & 0.0000 & -0.002 & 0.0000 \\
0.0000 & -0.263 & -0.011 & -0.006 & 0.0734 & 0.0064 & 0.0010 & -0.003 & 0.0010 & 0.0007 & -0.001 \\
0.0000 & 0.1345 & -0.222 & 0.0588 & -0.212 & 0.0042 & -0.006 & -0.018 & -0.002 & -0.021 & 0.0050 \\
0.0000 & -0.036 & 0.0160 & -0.304 & -0.324 & -0.036 & -0.004 & -0.114 & -0.005 & 0.0139 & -0.007 \\
0.0000 & 0.0123 & -0.022 & -0.016 & -0.152 & 0.0149 & 0.0017 & 0.0639 & 0.0024 & -0.006 & 0.0094 \\
0.0000 & -0.282 & -0.051 & 0.3264 & 0.6099 & -0.202 & 0.0027 & -0.104 & -0.004 & -0.001 & -0.026 \\
0.0000 & 1.1597 & -0.071 & -0.053 & 0.2303 & 0.0164 & -0.330 & -0.117 & 0.0013 & 0.0757 & -0.055 \\
0.0000 & -0.096 & 0.1826 & -0.002 & -0.294 & 0.0086 & -0.012 & -0.235 & -0.003 & -0.019 & -0.001 \\
0.0000 & -8.193 & -0.230 & -0.635 & -1.354 & -0.015 & 0.1368 & 2.6755 & -0.234 & 0.0155 & 0.5853 \\
0.0000 & -0.620 & -0.071 & 0.1693 & 0.2812 & 0.0220 & 0.0549 & 0.4024 & -0.005 & -0.319 & 0.1784 \\
0.0000 & -0.856 & -0.029 & 0.0251 & -0.026 & -0.006 & 0.0277 & 0.6958 & -0.007 & -0.085 & -0.075
\end{pmatrix}
\begin{pmatrix}
HFMD_{t-2} \\ WIN_{t-2} \\ SUN_{t-2} \\ PRES_{t-2} \\ TM_{t-2} \\ HUMID_{t-2} \\ RAIN_{t-2} \\ DTR_{t-2} \\ PM10_{t-2} \\ SO2_{t-2} \\ NO2_{t-2}
\end{pmatrix}
$$

CVAR_②:

$$
\begin{pmatrix}
HFMD_t \\ WIN_t \\ SUN_t \\ PRES_t \\ TM_t \\ HUMID_t \\ RAIN_t \\ DTR_t \\ PM10_t \\ SO2_t \\ NO2_t
\end{pmatrix}
=
\begin{pmatrix}
-0.001 \\ 0.0015 \\ -0.006 \\ 0.0194 \\ -0.015 \\ -0.014 \\ -0.047 \\ -0.003 \\ 0.0817 \\ -0.002 \\ 0.0462
\end{pmatrix}
+
$$

$$\begin{pmatrix} -0.212 & -0.042 & 0.0000 & 0.0000 & -0.063 & 0.0000 & 0.0000 & 0.0109 & 0.0000 & 0.0000 & 0.0000 \\ 0.0000 & -0.562 & -0.006 & -0.015 & 0.0565 & 10.003 & 0.0028 & -0.001 & 0.0011 & 0.0027 & -0.001 \\ 0.0000 & 0.0655 & -0.557 & 0.0920 & -0.192 & -0.023 & -0.004 & 0.0139 & -0.004 & 0.0061 & 0.0091 \\ 0.0000 & 0.9916 & -0.103 & 0.0327 & 0.0285 & 0.0051 & -0.001 & -0.035 & -0.004 & 0.0111 & -0.011 \\ 0.0000 & -0.280 & -0.012 & -0.055 & -0.007 & 0.0476 & -0.002 & 0.1189 & 0.0023 & 0.0034 & -0.002 \\ 0.0000 & -0.084 & -0.082 & -0.076 & 0.1338 & -0.320 & 0.0073 & -0.098 & -0.004 & -0.064 & 0.0201 \\ 0.0000 & 2.1235 & -0.230 & -0.331 & 0.2517 & 0.0085 & -0.429 & 0.0664 & -0.004 & -0.025 & 0.0495 \\ 0.0000 & 0.1166 & 0.1405 & 0.0806 & -0.232 & -0.021 & -0.007 & -0.464 & -0.008 & 0.0169 & 0.0202 \\ 0.0000 & -19.93 & -0.430 & -0.919 & 0.1394 & -0.432 & -0.014 & 3.1811 & -0.261 & -0.644 & 0.5548 \\ 0.0000 & -1.823 & 0.0469 & -0.428 & -0.254 & -0.104 & -0.001 & 0.4252 & 0.0081 & -0.433 & 0.1426 \\ 0.0000 & -2.895 & 0.2068 & 0.0554 & -0.156 & 0.0454 & -0.020 & 0.9532 & -0.012 & -0.074 & -0.184 \end{pmatrix} \begin{pmatrix} HFMD_{t-1} \\ WIN_{t-1} \\ SUN_{t-1} \\ PRES_{t-1} \\ TM_{t-1} \\ HUMID_{t-1} \\ RAIN_{t-1} \\ DTR_{t-1} \\ PM10_{t-1} \\ SO2_{t-1} \\ NO2_{t-1} \end{pmatrix} +$$

$$\begin{pmatrix} -0.117 & 0.0000 & 0.0283 & 0.0000 & -0.083 & 0.0000 & -0.004 & 0.0003 & 0.0000 & 0.0000 & 0.0012 \\ 0.0000 & -0.229 & -0.007 & -0.008 & 0.0810 & 20.005 & 0.0000 & -0.001 & 0.0005 & 0.0011 & 0.0004 \\ 0.0000 & 0.0680 & -0.224 & 0.0116 & -0.121 & 0.0023 & -0.010 & -0.039 & -0.002 & -0.034 & 0.0051 \\ 0.0000 & -0.110 & -0.014 & -0.327 & -0.244 & -0.035 & -0.002 & -0.101 & -0.005 & 0.0131 & -0.015 \\ 0.0000 & -0.003 & 0.0046 & -0.019 & -0.114 & 0.0160 & 0.0018 & 0.0351 & 0.0029 & -0.010 & 0.0113 \\ 0.0000 & -0.018 & -0.066 & 0.3712 & 0.3007 & -0.205 & 0.0042 & -0.013 & -0.002 & -0.010 & -0.008 \\ 0.0000 & 1.6548 & -0.152 & 0.0228 & 0.1193 & 0.0039 & -0.346 & -0.056 & -0.002 & 0.0560 & -0.036 \\ 0.0000 & -0.137 & 0.1788 & -0.024 & -0.260 & -0.001 & -0.013 & -0.267 & -0.003 & -0.030 & 0.0035 \\ 0.0000 & -5.101 & 0.1131 & -0.102 & -1.707 & 0.0253 & 0.1185 & 2.4691 & -0.218 & 0.0959 & 0.6037 \\ 0.0000 & 0.0991 & 0.0433 & 0.1595 & 0.2677 & 0.0203 & 0.0500 & 0.3011 & -0.004 & -0.321 & 0.1752 \\ 0.0000 & -0.248 & 0.1072 & 0.0056 & -0.386 & -0.038 & 0.0035 & 0.6458 & -0.009 & -0.118 & -0.035 \end{pmatrix} \begin{pmatrix} HFMD_{t-2} \\ WIN_{t-2} \\ SUN_{t-2} \\ PRES_{t-2} \\ TM_{t-2} \\ HUMID_{t-2} \\ RAIN_{t-2} \\ DTR_{t-2} \\ PM10_{t-2} \\ SO2_{t-2} \\ NO2_{t-2} \end{pmatrix}$$

CVAR_③:

$$\begin{pmatrix} HFMD_t \\ WIN_t \\ SUN_t \\ PRES_t \\ TM_t \\ HUMID_t \\ RAIN_t \\ DTR_t \\ PM10_t \\ SO2_t \\ NO2_t \end{pmatrix} = \begin{pmatrix} 0.0041 \\ -0.002 \\ 0.2475 \\ -0.021 \\ 0.0215 \\ 0.0011 \\ -0.015 \\ 0.0149 \\ -0.077 \\ -0.017 \\ -0.018 \end{pmatrix} +$$

$$
\begin{pmatrix}
-0.232 & 0.0000 & 0.0000 & 0.0000 & -0.025 & 0.0000 & 0.0000 & 0.0107 & 0.0000 & -0.004 & 0.0000 \\
0.0000 & -0.550 & -0.011 & -0.009 & 0.0692 & 0.0052 & 0.0028 & -0.002 & 0.0011 & 0.0024 & 0.0002 \\
0.0000 & 0.2475 & -0.549 & 0.0753 & -0.186 & -0.021 & -0.010 & 0.0028 & -0.005 & -0.003 & 0.0178 \\
0.0000 & 1.0129 & -0.120 & 0.0428 & 0.1492 & 0.0121 & -0.004 & -0.015 & -0.003 & 0.0126 & -0.017 \\
0.0000 & -0.238 & -0.006 & -0.066 & -0.059 & 0.0385 & 0.0022 & 0.1088 & 0.0021 & 0.0001 & 0.0007 \\
0.0000 & -0.321 & -0.124 & -0.075 & 0.0265 & -0.327 & 0.0006 & -0.033 & -0.003 & -0.054 & -0.000 \\
0.0000 & 1.4139 & -0.149 & -0.205 & 0.4243 & 0.0350 & -0.438 & -0.001 & -0.003 & -0.011 & 0.0328 \\
0.0000 & 0.2872 & 0.1624 & 0.0411 & -0.263 & -0.018 & -0.006 & -0.477 & -0.008 & 0.0078 & 0.0283 \\
0.0000 & -20.37 & 0.1298 & -0.996 & -1.558 & -0.486 & 0.1464 & 2.9798 & -0.266 & -0.569 & 0.4782 \\
0.0000 & -1.683 & 0.0834 & -0.488 & -0.605 & -0.117 & 0.0221 & 0.4551 & 0.0118 & -0.461 & 0.1404 \\
0.0000 & -2.748 & 0.1333 & -0.092 & -0.518 & 0.0284 & 0.0004 & 1.1158 & -0.014 & -0.064 & -0.227
\end{pmatrix}
\begin{pmatrix}
HFMD_{t-1} \\ WIN_{t-1} \\ SUN_{t-1} \\ PRES_{t-1} \\ TM_{t-1} \\ HUMID_{t-1} \\ RAIN_{t-1} \\ DTR_{t-1} \\ PM10_{t-1} \\ SO2_{t-1} \\ NO2_{t-1}
\end{pmatrix} +
$$

$$
\begin{pmatrix}
-0.075 & 0.0000 & 0.0310 & 0.0039 & -0.114 & -0.015 & 0.0000 & 0.0000 & 0.0000 & -0.003 & -0.002 \\
0.0000 & -0.210 & -0.002 & -0.010 & 0.0736 & 0.0047 & 0.0008 & -0.007 & 0.0003 & 0.0003 & 0.0023 \\
0.0000 & 0.1809 & -0.230 & 0.0135 & -0.098 & -0.004 & -0.008 & -0.026 & -0.004 & -0.037 & 0.0115 \\
0.0000 & -0.128 & -0.005 & -0.336 & -0.225 & -0.050 & -0.002 & -0.123 & -0.005 & 0.0026 & -0.011 \\
0.0000 & 0.0566 & -0.001 & -0.019 & -0.134 & 0.0155 & 0.0029 & 0.0380 & 0.0024 & -0.008 & 0.0069 \\
0.0000 & -0.413 & -0.092 & 0.3916 & 0.3594 & -0.191 & 0.0074 & 0.0594 & -0.000 & -0.003 & 0.0002 \\
0.0000 & 0.6506 & 0.1339 & -0.020 & 0.2667 & -0.000 & -0.361 & -0.219 & -0.004 & 0.0490 & 0.0092 \\
0.0000 & -0.021 & 0.1789 & -0.032 & -0.280 & -0.007 & -0.010 & -0.261 & -0.004 & -0.032 & 0.0035 \\
0.0000 & -4.730 & 0.2181 & -0.048 & -1.914 & 0.0693 & 0.1526 & 3.2509 & -0.191 & 0.1372 & 0.3981 \\
0.0000 & 0.4651 & -0.112 & 0.2299 & 0.3846 & 0.0568 & 0.0439 & 0.5228 & 0.0004 & -0.2933 & 0.1154 \\
0.0000 & -0.107 & -0.026 & 0.0348 & -0.626 & -0.020 & -0.005 & 0.8557 & -0.003 & -0.0956 & -0.092
\end{pmatrix}
\begin{pmatrix}
HFMD_{t-2} \\ WIN_{t-2} \\ SUN_{t-2} \\ PRES_{t-2} \\ TM_{t-2} \\ HUMID_{t-2} \\ RAIN_{t-2} \\ DTR_{t-2} \\ PM10_{t-2} \\ SO2_{t-2} \\ NO2_{t-2}
\end{pmatrix}
$$

CVAR_④:

$$
\begin{pmatrix}
HFMD_t \\ WIN_t \\ SUN_t \\ PRES_t \\ TM_t \\ HUMID_t \\ RAIN_t \\ DTR_t \\ PM10_t \\ SO2_t \\ NO2_t
\end{pmatrix} =
\begin{pmatrix}
-0.007 \\ 0.0037 \\ 0.0014 \\ 0.0232 \\ -0.014 \\ -0.022 \\ 0.0066 \\ 0.0023 \\ -0.037 \\ -0.022 \\ -0.005
\end{pmatrix} +
$$

$$\begin{pmatrix} -0.266 & 0.0000 & 0.0000 & 0.0000 & -0.038 & -0.002 & 0.0000 & 0.0000 & 0.0000 & -0.006 & 0.0000 \\ 0.0000 & -0.581 & -0.013 & -0.016 & 0.0495 & 10.002 & 0.0030 & 0.0015 & 0.0009 & 0.0030 & -0.001 \\ 0.0000 & 0.3137 & -0.551 & 0.0665 & -0.116 & -0.017 & -0.013 & -0.056 & -0.007 & -0.005 & 0.0240 \\ 0.0000 & 1.0825 & -0.110 & 0.0631 & 0.2387 & 0.0025 & 0.0004 & -0.066 & -0.004 & 0.0118 & -0.003 \\ 0.0000 & -0.291 & -0.003 & -0.073 & -0.097 & 10.041 & 0.0003 & 0.1003 & 0.0018 & 0.0035 & -0.000 \\ 0.0000 & -0.444 & -0.107 & -0.115 & -0.184 & -0.385 & 0.0018 & 0.0059 & -0.000 & -0.067 & -0.001 \\ 0.0000 & 1.0361 & -0.036 & -0.188 & 0.2451 & 0.0313 & -0.443 & -0.052 & -0.000 & 0.0045 & 0.0131 \\ 0.0000 & 0.3445 & 0.2045 & 0.0271 & -0.202 & -0.006 & -0.009 & -0.567 & -0.009 & 0.0097 & 0.0346 \\ 0.0000 & -18.16 & 0.2710 & -1.252 & -4.224 & -0.630 & 0.0704 & 2.9455 & -0.272 & -0.545 & 0.5364 \\ 0.0000 & -1.579 & 0.1053 & -0.412 & -0.623 & -0.086 & 0.0158 & 0.5069 & 0.0082 & -0.461 & 0.1297 \\ 0.0000 & -2.172 & 0.3953 & -0.246 & -0.948 & -0.016 & -0.014 & 0.9064 & -0.019 & -0.068 & -0.185 \end{pmatrix} \begin{pmatrix} HFMD_{t-1} \\ WIN_{t-1} \\ SUN_{t-1} \\ PRES_{t-1} \\ TM_{t-1} \\ HUMID_{t-1} \\ RAIN_{t-1} \\ DTR_{t-1} \\ PM10_{t-1} \\ SO2_{t-1} \\ NO2_{t-1} \end{pmatrix} +$$

$$\begin{pmatrix} -0.104 & -0.124 & 0.0156 & 0.0000 & -0.107 & -0.013 & 0.0000 & 0.0000 & -0.001 & -0.003 & -0.004 \\ 0.0000 & -0.251 & -0.010 & -0.011 & 0.0655 & 20.002 & 0.0010 & 0.0014 & 0.0003 & -0.001 & 0.0024 \\ 0.0000 & 0.2355 & -0.211 & 0.0308 & -0.034 & 0.0054 & -0.006 & -0.068 & -0.005 & -0.034 & 0.0121 \\ 0.0000 & -0.146 & 0.0216 & -0.329 & -0.302 & -0.063 & 0.0027 & -0.168 & -0.005 & -0.001 & -0.006 \\ 0.0000 & 0.1045 & 0.0143 & -0.003 & -0.077 & 20.028 & 0.0029 & 0.0332 & 0.0019 & -0.007 & 0.0086 \\ 0.0000 & -0.638 & -0.195 & 0.2723 & -0.024 & -0.258 & -0.001 & 0.1437 & 0.0079 & -0.000 & -0.017 \\ 0.0000 & 0.6036 & 0.1392 & -0.079 & 0.3365 & 0.0146 & -0.356 & -0.134 & -0.001 & 0.0420 & 0.0239 \\ 0.0000 & 0.0780 & 0.2247 & -0.009 & -0.147 & 0.0076 & -0.010 & -0.340 & -0.006 & -0.026 & 0.0112 \\ 0.0000 & -4.799 & 0.5329 & -0.407 & -2.661 & 0.0731 & 0.0559 & 2.4938 & -0.182 & 0.2298 & 0.3465 \\ 0.0000 & 0.2479 & -0.137 & 0.1223 & 0.3395 & 0.0381 & 0.0340 & 0.4473 & -0.001 & -0.281 & 0.1103 \\ 0.0000 & 0.0426 & 0.1634 & -0.053 & -0.937 & -0.017 & -0.029 & 0.6418 & -0.004 & -0.077 & -0.086 \end{pmatrix} \begin{pmatrix} HFMD_{t-2} \\ WIN_{t-2} \\ SUN_{t-2} \\ PRES_{t-2} \\ TM_{t-2} \\ HUMID_{t-2} \\ RAIN_{t-2} \\ DTR_{t-2} \\ PM10_{t-2} \\ SO2_{t-2} \\ NO2_{t-2} \end{pmatrix}$$

CVAR_⑤:

$$\begin{pmatrix} HFMD_t \\ WIN_t \\ SUN_t \\ PRES_t \\ TM_t \\ HUMID_t \\ RAIN_t \\ DTR_t \\ PM10_t \\ SO2_t \\ NO2_t \end{pmatrix} = \begin{pmatrix} 0.0063 \\ 0.0003 \\ -0.005 \\ -0.010 \\ 0.0211 \\ 0.0475 \\ 0.0347 \\ -0.001 \\ -0.029 \\ -0.050 \\ -0.013 \end{pmatrix} +$$

$$\begin{pmatrix} -0.253 & 0.0000 & 0.0000 & 0.0201 & 0.0000 & 0.0000 & 0.0000 & 0.0000 & 0.0000 & -0.004 & 0.0000 \\ 0.0000 & -0.582 & -0.020 & -0.007 & 0.0504 & 10.003 & 0.0024 & 0.0091 & 0.0008 & 0.0028 & -0.001 \\ 0.0000 & 0.1105 & -0.524 & 0.0168 & -0.185 & -0.006 & -0.014 & -0.047 & -0.006 & -0.019 & 0.0238 \\ 0.0000 & 1.0168 & -0.117 & 0.0920 & 0.1671 & 0.0011 & -0.007 & -0.059 & -0.007 & 0.0093 & -0.003 \\ 0.0000 & -0.331 & 0.0278 & -0.067 & -0.093 & 0.0456 & 0.0031 & 0.0815 & 0.0017 & 0.0017 & 0.0020 \\ 0.0000 & -0.435 & -0.171 & -0.071 & -0.042 & -0.405 & 0.0123 & -0.004 & 0.0051 & -0.029 & -0.015 \\ 0.0000 & 0.7091 & -0.082 & -0.179 & 0.1217 & 0.0492 & -0.459 & 0.0055 & -0.001 & 0.0560 & -0.012 \\ 0.0000 & 0.1280 & 0.2332 & -0.034 & -0.370 & -0.010 & -0.011 & -0.591 & -0.008 & -0.005 & 0.0409 \\ 0.0000 & -14.41 & 1.1719 & -1.270 & -4.032 & -0.564 & 0.1214 & 2.4327 & -0.260 & -0.478 & 0.6264 \\ 0.0000 & -0.986 & 0.3681 & -0.238 & -0.588 & -0.019 & 0.0248 & 0.4003 & 0.0087 & -0.447 & 0.1448 \\ 0.0000 & -1.062 & 0.6947 & -0.234 & -0.908 & -0.006 & 0.0027 & 0.7014 & -0.020 & -0.035 & -0.140 \end{pmatrix} \begin{pmatrix} HFMD_{t-1} \\ WIN_{t-1} \\ SUN_{t-1} \\ PRES_{t-1} \\ TM_{t-1} \\ HUMID_{t-1} \\ RAIN_{t-1} \\ DTR_{t-1} \\ PM10_{t-1} \\ SO2_{t-1} \\ NO2_{t-1} \end{pmatrix} +$$

$$\begin{pmatrix} -0.102 & -0.166 & 0.0129 & 0.0001 & -0.122 & -0.019 & -0.005 & 0.0000 & -0.001 & 0.0000 & -0.004 \\ 0.0000 & -0.292 & -0.011 & -0.013 & 0.0639 & 20.003 & 0.0009 & 0.0045 & 0.0001 & 0.0012 & 0.0010 \\ 0.0000 & 0.1509 & -0.187 & 0.0287 & -0.085 & 0.0223 & -0.010 & -0.069 & -0.005 & -0.026 & 0.0126 \\ 0.0000 & -0.088 & 0.0306 & -0.313 & -0.386 & -0.068 & 0.0012 & -0.147 & -0.005 & 0.0036 & 0.0060 \\ 0.0000 & 0.0631 & 0.0249 & -0.020 & -0.116 & 0.0319 & 0.0020 & 0.0317 & 0.0022 & -0.008 & 0.0071 \\ 0.0000 & -0.560 & -0.238 & 0.3262 & 0.1628 & -0.254 & 0.0076 & 0.1051 & 0.0088 & -0.008 & -0.023 \\ 0.0000 & 0.0847 & 0.1292 & -0.163 & 0.4411 & 0.0333 & -0.365 & -0.021 & -0.004 & 0.0689 & -0.014 \\ 0.0000 & -0.043 & 0.2319 & -0.004 & -0.156 & 0.0142 & -0.009 & -0.369 & -0.005 & -0.024 & 0.0159 \\ 0.0000 & -1.900 & 0.4848 & 0.1134 & -2.263 & 0.0443 & 0.0396 & 2.0989 & -0.178 & 0.3257 & 0.2679 \\ 0.0000 & 0.4041 & -0.056 & 0.1072 & 0.3202 & 0.0627 & 0.0257 & 0.3940 & 0.0005 & -0.238 & 0.0647 \\ 0.0000 & 0.2936 & 0.3025 & 0.0224 & -0.981 & -0.003 & -0.040 & 0.4725 & -0.001 & -0.064 & -0.143 \end{pmatrix} \begin{pmatrix} HFMD_{t-2} \\ WIN_{t-2} \\ SUN_{t-2} \\ PRES_{t-2} \\ TM_{t-2} \\ HUMID_{t-2} \\ RAIN_{t-2} \\ DTR_{t-2} \\ PM10_{t-2} \\ SO2_{t-2} \\ NO2_{t-2} \end{pmatrix}$$

CVAR_⑥:

$$\begin{pmatrix} HFMD_t \\ WIN_t \\ SUN_t \\ PRES_t \\ TM_t \\ HUMID_t \\ RAIN_t \\ DTR_t \\ PM10_t \\ SO2_t \\ NO2_t \end{pmatrix} = \begin{pmatrix} -0.010 \\ 0.0002 \\ -0.002 \\ 0.0195 \\ -0.012 \\ -0.021 \\ 0.0059 \\ -0.001 \\ -0.049 \\ -0.009 \\ -0.013 \end{pmatrix} +$$

$$
\begin{pmatrix}
-0.3261 & 0.0668 & 0.0000 & 0.0229 & -0.041 & 0.0000 & 0.0000 & 0.0000 & 0.0000 & 0.0000 & 0.0000 \\
0.0000 & -0.541 & -0.016 & -0.008 & 0.0485 & 10.004 & 0.0017 & 0.0105 & 0.0002 & 0.0032 & -0.001 \\
0.0000 & 0.0387 & -0.516 & 0.0165 & -0.217 & 0.0042 & -0.015 & -0.028 & -0.008 & -0.013 & 0.0193 \\
0.0000 & 1.2107 & -0.098 & 0.1838 & 0.2403 & 0.0252 & -0.014 & -0.077 & -0.006 & -0.004 & 0.0007 \\
0.0000 & -0.371 & 0.0322 & -0.107 & -0.113 & 10.047 & 0.0031 & 0.0775 & 0.0001 & 0.0034 & 0.0044 \\
0.0000 & -1.243 & -0.114 & -0.044 & -0.260 & -0.469 & 0.0233 & -0.075 & 0.0190 & 0.0152 & -0.053 \\
0.0000 & 0.5835 & -0.084 & -0.150 & -0.016 & 0.0195 & -0.473 & 0.0012 & -0.002 & 0.1490 & -0.024 \\
0.0000 & 0.1791 & 0.2549 & -0.030 & -0.358 & -0.006 & -0.012 & -0.607 & -0.007 & -0.013 & 0.0413 \\
0.0000 & -12.22 & 1.1145 & -0.171 & -2.622 & -0.663 & 0.1638 & 1.9066 & -0.287 & -0.348 & 0.8022 \\
0.0000 & -0.914 & 0.3195 & -0.177 & -0.587 & -0.079 & 0.0165 & 0.2013 & 0.0024 & -0.473 & 0.1488 \\
0.0000 & -2.189 & 0.7748 & -0.264 & -1.295 & -0.101 & 0.0165 & 0.4561 & -0.014 & -0.001 & -0.173
\end{pmatrix}
\begin{pmatrix}
HFMD_{t-1} \\ WIN_{t-1} \\ SUN_{t-1} \\ PRES_{t-1} \\ TM_{t-1} \\ HUMID_{t-1} \\ RAIN_{t-1} \\ DTR_{t-1} \\ PM10_{t-1} \\ SO2_{t-1} \\ NO2_{t-1}
\end{pmatrix} +
$$

$$
\begin{pmatrix}
-0.122 & -0.076 & 0.0249 & 0.0132 & -0.079 & 0.0000 & 0.0000 & 0.0000 & -0.002 & 0.0000 & 0.0000 \\
0.0000 & -0.275 & -0.006 & -0.005 & 0.0627 & 20.003 & 0.0007 & 0.0016 & 0.0001 & 0.0032 & 0.0007 \\
0.0000 & 0.1206 & -0.208 & 0.0233 & -0.078 & 0.0207 & -0.012 & -0.039 & -0.004 & -0.031 & 0.0076 \\
0.0000 & 0.1732 & 0.0816 & -0.272 & -0.357 & -0.050 & -0.005 & -0.165 & -0.006 & -0.008 & 0.0118 \\
0.0000 & 0.0184 & 0.0335 & -0.031 & -0.078 & 20.036 & 0.0017 & 0.0288 & 0.0027 & -0.010 & 0.0031 \\
0.0000 & -1.009 & -0.194 & 0.3036 & 0.1688 & -0.255 & 0.0229 & -0.015 & 0.0095 & 0.0510 & -0.018 \\
0.0000 & -0.087 & 0.0946 & -0.129 & 0.4787 & 0.0469 & -0.359 & 0.0011 & -0.004 & 0.1522 & -0.007 \\
0.0000 & -0.006 & 0.1741 & 0.0135 & -0.122 & 0.0071 & -0.011 & -0.347 & -0.003 & -0.040 & 0.0136 \\
0.0000 & 0.8135 & -0.521 & 0.8099 & -1.124 & -0.029 & 0.0018 & 1.9628 & -0.056 & -0.180 & 0.1685 \\
0.0000 & 0.2712 & -0.246 & 0.0527 & 0.2541 & -0.036 & 0.0142 & 0.3344 & 0.0150 & -0.257 & 0.0186 \\
0.0000 & 0.3019 & 0.2109 & 0.0096 & -0.853 & -0.004 & -0.048 & 0.4197 & 0.0105 & -0.066 & -0.168
\end{pmatrix}
\begin{pmatrix}
HFMD_{t-2} \\ WIN_{t-2} \\ SUN_{t-2} \\ PRES_{t-2} \\ TM_{t-2} \\ HUMID_{t-2} \\ RAIN_{t-2} \\ DTR_{t-2} \\ PM10_{t-2} \\ SO2_{t-2} \\ NO2_{t-2}
\end{pmatrix}
$$

CVAR_⑦:

$$
\begin{pmatrix}
HFMD_t \\ WIN_t \\ SUN_t \\ PRES_t \\ TM_t \\ HUMID_t \\ RAIN_t \\ DTR_t \\ PM10_t \\ SO2_t \\ NO2_t
\end{pmatrix} =
\begin{pmatrix}
0.0054 \\ -0.001 \\ 0.0061 \\ -0.013 \\ 0.0214 \\ 0.0125 \\ 0.0083 \\ 0.0026 \\ -0.130 \\ -0.015 \\ -0.046
\end{pmatrix} +
$$

$$\begin{pmatrix} -0.253 & 0.1166 & 0.0000 & 0.0356 & 0.0000 & 0.0000 & 0.0000 & 0.0000 & 0.0000 & 0.0000 & 0.0000 \\ 0.0000 & -0.531 & -0.013 & -0.005 & 0.0501 & 10.007 & -0.001 & 0.0097 & 0.0001 & 0.0004 & 0.0006 \\ 0.0000 & -0.092 & -0.463 & 0.0741 & -0.213 & 0.0134 & -0.024 & -0.073 & -0.007 & -0.000 & 0.0134 \\ 0.0000 & 1.1836 & -0.081 & 0.1618 & 0.1273 & 0.0250 & -0.020 & -0.079 & -0.007 & -0.017 & 0.0071 \\ 0.0000 & -0.371 & 0.0370 & -0.105 & -0.122 & 10.040 & 0.0097 & 0.0761 & 0.0000 & 0.0061 & 0.0073 \\ 0.0000 & -1.423 & -0.202 & -0.031 & 0.0482 & -0.443 & 0.0185 & -0.021 & 0.0190 & 0.0274 & -0.078 \\ 0.0000 & -0.013 & -0.102 & -0.051 & -0.072 & 0.0391 & -0.627 & 0.0498 & -0.002 & 0.0789 & -0.043 \\ 0.0000 & -0.084 & 0.2978 & -0.024 & -0.390 & -0.007 & -0.009 & -0.658 & -0.005 & -0.016 & 0.0378 \\ 0.0000 & -15.25 & 1.1791 & -0.252 & -2.921 & -0.824 & 0.2275 & 1.1754 & -0.304 & -0.295 & 0.7935 \\ 0.0000 & -0.867 & 0.2689 & -0.103 & -0.419 & -0.094 & 0.0469 & 0.1630 & -0.000 & -0.488 & 0.1431 \\ 0.0000 & -2.733 & 0.7679 & -0.291 & -1.310 & -0.138 & 0.0215 & 0.3540 & -0.021 & 0.0696 & -0.192 \end{pmatrix} \begin{pmatrix} HFMD_{t-1} \\ WIN_{t-1} \\ SUN_{t-1} \\ PRES_{t-1} \\ TM_{t-1} \\ HUMID_{t-1} \\ RAIN_{t-1} \\ DTR_{t-1} \\ PM10_{t-1} \\ SO2_{t-1} \\ NO2_{t-1} \end{pmatrix} +$$

$$\begin{pmatrix} -0.113 & -0.143 & 0.0113 & 0.0259 & -0.088 & 0.0000 & 0.0000 & 0.0000 & -0.001 & 0.0000 & -0.003 \\ 0.0000 & -0.274 & -0.006 & -0.001 & 0.0633 & 20.004 & 0.0027 & 0.0038 & 0.0006 & -0.003 & 0.0022 \\ 0.0000 & -0.149 & -0.187 & -0.012 & -0.196 & 0.0264 & -0.020 & -0.047 & -0.005 & -0.001 & 0.0056 \\ 0.0000 & 0.0285 & 0.0631 & -0.289 & -0.341 & -0.042 & -0.007 & -0.144 & -0.006 & -0.027 & 0.0131 \\ 0.0000 & 0.0203 & 0.0293 & -0.020 & -0.079 & 20.040 & 0.0030 & 0.0376 & 0.0017 & 0.0027 & 0.0018 \\ 0.0000 & -1.129 & -0.268 & 0.2711 & 0.2806 & -0.272 & 0.0234 & -0.001 & 0.0098 & 0.0604 & -0.033 \\ 0.0000 & -1.006 & 0.1039 & -0.141 & 0.5718 & 0.0589 & -0.366 & 0.0646 & 0.0003 & 0.0231 & 0.0376 \\ 0.0000 & -0.314 & 0.2086 & -0.038 & -0.245 & 0.0055 & -0.012 & -0.383 & -0.004 & -0.010 & 0.0104 \\ 0.0000 & -3.106 & 0.3048 & 0.4708 & -1.535 & -0.143 & 0.0678 & 0.9688 & -0.086 & -0.091 & 0.1962 \\ 0.0000 & 0.2657 & -0.092 & 0.0496 & 0.0637 & -0.057 & 0.0144 & 0.1668 & 0.0081 & -0.239 & 0.0288 \\ 0.0000 & -0.223 & 0.4068 & -0.074 & -0.908 & -0.025 & -0.037 & 0.1962 & -0.001 & 0.0161 & -0.167 \end{pmatrix} \begin{pmatrix} HFMD_{t-2} \\ WIN_{t-2} \\ SUN_{t-2} \\ PRES_{t-2} \\ TM_{t-2} \\ HUMID_{t-2} \\ RAIN_{t-2} \\ DTR_{t-2} \\ PM10_{t-2} \\ SO2_{t-2} \\ NO2_{t-2} \end{pmatrix}$$

CVAR_⑧:

$$\begin{pmatrix} HFMD_t \\ WIN_t \\ SUN_t \\ PRES_t \\ TM_t \\ HUMID_t \\ RAIN_t \\ DTR_t \\ PM10_t \\ SO2_t \\ NO2_t \end{pmatrix} = \begin{pmatrix} -0.009 \\ 0.0023 \\ -0.001 \\ 0.0058 \\ -0.015 \\ -0.005 \\ 0.0115 \\ -0.001 \\ -0.019 \\ -0.002 \\ -0.021 \end{pmatrix} +$$

$$\begin{pmatrix} -0.269 & 0.1126 & 0.0193 & 0.0000 & -0.049 & 0.0000 & 0.0000 & 0.0000 & 0.0000 & 0.0000 & 0.0000 \\ 0.0000 & -0.574 & -0.012 & -0.007 & 0.0447 & 10.005 & -0.000 & 0.0097 & 0.0006 & 0.0023 & -0.003 \\ 0.0000 & -0.114 & -0.446 & 0.0569 & -0.251 & 0.0191 & -0.024 & -0.092 & -0.006 & 0.0104 & 0.0166 \\ 0.0000 & 1.1914 & -0.061 & 0.1761 & 0.0748 & 0.0357 & -0.023 & -0.110 & -0.013 & 0.0060 & 0.0114 \\ 0.0000 & -0.374 & 0.0288 & -0.123 & -0.165 & 0.0393 & 0.0103 & 0.0968 & 0.0002 & 0.0090 & 0.0103 \\ 0.0000 & -1.136 & -0.119 & 0.0371 & 0.1689 & -0.448 & 0.0237 & -0.074 & 0.0285 & -0.000 & -0.064 \\ 0.0000 & -0.039 & -0.032 & -0.006 & -0.161 & 0.0270 & -0.629 & -0.024 & 0.0043 & 0.1082 & -0.049 \\ 0.0000 & -0.354 & 0.3069 & 0.0131 & -0.352 & 0.0094 & -0.009 & -0.686 & -0.005 & -0.019 & 0.0364 \\ 0.0000 & -14.83 & 1.3596 & -0.287 & -2.642 & -0.729 & 0.2297 & 1.1441 & -0.279 & -0.561 & 0.9645 \\ 0.0000 & -0.946 & 0.1963 & -0.053 & -0.158 & -0.094 & 0.0367 & 0.1396 & -0.005 & -0.500 & 0.1145 \\ 0.0000 & -3.524 & 0.8251 & -0.139 & -0.816 & -0.111 & 0.0237 & 0.2269 & -0.023 & 0.0135 & -0.153 \end{pmatrix} \begin{pmatrix} HFMD_{t-1} \\ WIN_{t-1} \\ SUN_{t-1} \\ PRES_{t-1} \\ TM_{t-1} \\ HUMID_{t-1} \\ RAIN_{t-1} \\ DTR_{t-1} \\ PM10_{t-1} \\ SO2_{t-1} \\ NO2_{t-1} \end{pmatrix} +$$

$$\begin{pmatrix} -0.160 & -0.162 & 0.0000 & 0.0000 & -0.095 & 0.0000 & 0.0000 & 0.0000 & -0.003 & 0.0000 & 0.0000 \\ 0.0000 & -0.304 & -0.008 & -0.008 & 0.0565 & 20.003 & 0.0024 & 0.0050 & 0.0001 & -0.002 & 0.0013 \\ 0.0000 & -0.225 & -0.216 & -0.004 & -0.262 & 0.0346 & -0.021 & -0.034 & -0.001 & -0.007 & 0.0038 \\ 0.0000 & 0.1183 & 0.0858 & -0.261 & -0.347 & -0.016 & -0.010 & -0.129 & -0.010 & -0.018 & 0.0301 \\ 0.0000 & 0.1079 & -0.001 & -0.014 & -0.056 & 0.0429 & 0.0035 & 0.0698 & 0.0024 & 0.0012 & 0.0003 \\ 0.0000 & -0.775 & -0.123 & 0.2133 & 0.2691 & -0.293 & 0.0210 & -0.194 & 0.0070 & 0.0765 & -0.040 \\ 0.0000 & -0.958 & 0.1586 & -0.196 & 0.5036 & 0.0546 & -0.366 & 0.0407 & -0.006 & 0.0554 & 0.0529 \\ 0.0000 & -0.604 & 0.1978 & 0.0065 & -0.274 & 0.0259 & -0.010 & -0.367 & 0.0013 & -0.027 & 0.0137 \\ 0.0000 & -0.677 & 0.1029 & 1.0100 & 0.3373 & 0.0919 & 0.0850 & 0.8707 & -0.062 & -0.140 & 0.1904 \\ 0.0000 & -0.161 & -0.128 & 0.0614 & 0.1999 & -0.051 & 0.0146 & 0.1517 & 0.0140 & -0.264 & 0.0001 \\ 0.0000 & -0.300 & 0.2980 & 0.0559 & -0.514 & -0.006 & -0.037 & 0.2267 & 0.0137 & -0.056 & -0.193 \end{pmatrix} \begin{pmatrix} HFMD_{t-2} \\ WIN_{t-2} \\ SUN_{t-2} \\ PRES_{t-2} \\ TM_{t-2} \\ HUMID_{t-2} \\ RAIN_{t-2} \\ DTR_{t-2} \\ PM10_{t-2} \\ SO2_{t-2} \\ NO2_{t-2} \end{pmatrix}$$

CVAR_⑨:

$$\begin{pmatrix} HFMD_t \\ WIN_t \\ SUN_t \\ PRES_t \\ TM_t \\ HUMID_t \\ RAIN_t \\ DTR_t \\ PM10_t \\ SO2_t \\ NO2_t \end{pmatrix} = \begin{pmatrix} 0.0004 \\ -0.002 \\ 0.0153 \\ -0.010 \\ 0.0252 \\ -0.020 \\ -0.005 \\ 0.0134 \\ -0.116 \\ 0.0061 \\ -0.008 \end{pmatrix} +$$

$$\begin{pmatrix} -0.279 & 0.0563 & 0.0035 & 0.0000 & -0.057 & -0.010 & -0.005 & 0.0000 & 0.0000 & 0.0059 & -0.005 \\ 0.0000 & -0.548 & -0.015 & -0.002 & 0.0528 & 0.0062 & -0.001 & 0.0148 & 0.0003 & -0.001 & -0.001 \\ 0.0000 & -0.276 & -0.418 & 0.0792 & -0.245 & 0.0217 & -0.017 & -0.082 & -0.002 & 0.0113 & -0.011 \\ 0.0000 & 1.1363 & -0.112 & 0.2077 & 0.0827 & 0.0372 & -0.019 & -0.042 & -0.013 & 0.0191 & 0.0002 \\ 0.0000 & -0.406 & 0.0389 & -0.123 & -0.197 & 0.0367 & 0.0111 & 0.0975 & 0.0004 & 0.0064 & 0.0077 \\ 0.0000 & -0.883 & -0.133 & 0.0174 & 0.2437 & -0.456 & 0.0037 & -0.153 & 0.0273 & 0.0203 & -0.043 \\ 0.0000 & 1.1975 & -0.240 & 0.0447 & -0.044 & 0.0310 & -0.661 & 0.1117 & -0.001 & 0.1105 & -0.023 \\ 0.0000 & -0.307 & 0.2233 & 0.0056 & -0.402 & 0.0044 & -0.003 & -0.582 & -0.003 & -0.001 & 0.0139 \\ 0.0000 & -13.73 & 2.0766 & -0.022 & -2.569 & -0.624 & 0.1050 & 0.3164 & -0.263 & -0.300 & 0.7874 \\ 0.0000 & -1.150 & 0.2931 & -0.083 & -0.187 & -0.104 & 0.0391 & -0.017 & -0.009 & -0.469 & 0.1067 \\ 0.0000 & -3.694 & 1.1125 & -0.113 & -0.858 & -0.108 & 0.0310 & -0.012 & -0.022 & 0.0518 & -0.165 \end{pmatrix} \begin{pmatrix} HFMD_{t-1} \\ WIN_{t-1} \\ SUN_{t-1} \\ PRES_{t-1} \\ TM_{t-1} \\ HUMID_{t-1} \\ RAIN_{t-1} \\ DTR_{t-1} \\ PM10_{t-1} \\ SO2_{t-1} \\ NO2_{t-1} \end{pmatrix} +$$

$$\begin{pmatrix} -0.157 & -0.131 & 0.0000 & 0.0000 & -0.139 & -0.015 & -0.006 & 0.0000 & -0.002 & 0.0065 & 0.0000 \\ 0.0000 & -0.300 & -0.009 & -0.011 & 0.0646 & 0.0046 & 0.0017 & 0.0063 & 0.0003 & -0.003 & -0.001 \\ 0.0000 & -0.337 & -0.178 & 0.0376 & -0.271 & 0.0398 & -0.016 & -0.032 & -0.001 & 0.0282 & -0.001 \\ 0.0000 & 0.2261 & 0.0253 & -0.248 & -0.261 & 0.0084 & -0.012 & -0.052 & -0.008 & -0.016 & 0.0189 \\ 0.0000 & 0.0792 & -0.011 & -0.029 & -0.050 & 0.0428 & 0.0052 & 0.0946 & 0.0011 & 0.0099 & 0.0007 \\ 0.0000 & -0.610 & -0.124 & 0.2042 & 0.3578 & -0.285 & 0.0047 & -0.287 & 0.0083 & 0.0490 & -0.041 \\ 0.0000 & 0.0451 & 0.0031 & -0.044 & 0.4328 & 0.0549 & -0.359 & 0.0616 & -0.001 & 0.0658 & -0.001 \\ 0.0000 & -0.526 & 0.1647 & 0.0260 & -0.345 & 0.0221 & -0.008 & -0.323 & 0.0009 & 0.0240 & 0.0111 \\ 0.0000 & -0.460 & 0.7365 & 0.9436 & 0.0499 & 0.0933 & 0.0359 & 0.5719 & -0.052 & -0.249 & 0.2157 \\ 0.0000 & -0.167 & 0.0588 & 0.1024 & -0.028 & -0.072 & 0.0066 & 0.0131 & 0.0149 & -0.314 & 0.0241 \\ 0.0000 & -0.575 & 0.4284 & 0.1104 & -0.337 & 0.0281 & -0.018 & 0.1536 & 0.0124 & -0.053 & -0.196 \end{pmatrix} \begin{pmatrix} HFMD_{t-2} \\ WIN_{t-2} \\ SUN_{t-2} \\ PRES_{t-2} \\ TM_{t-2} \\ HUMID_{t-2} \\ RAIN_{t-2} \\ DTR_{t-2} \\ PM10_{t-2} \\ SO2_{t-2} \\ NO2_{t-2} \end{pmatrix}$$
